# A randomized controlled trial study of a life review art intervention for older adults living alone

**DOI:** 10.3389/fpsyg.2025.1669119

**Published:** 2025-11-19

**Authors:** Pei-Ching Tsai, Wen-Huei Chou, Shu-Yi Liao

**Affiliations:** 1 Graduate School of Design, National Yunlin University of Science and Technology, Douliu, Taiwan; 2 Department of Physical Medicine and Rehabilitation, National Taiwan University Hospital Yunlin Branch, Yunlin, Taiwan

**Keywords:** older adults living, mental health, trauma, life review, art therapy, depression, adaptive behavior

## Abstract

**Background:**

Older adults living alone face high risks of depression and functional decline; culturally adapted, non-pharmacological options are needed.

**Objective:**

To test whether a 12-week Life Review Art Therapy (LRAT) program improves psychological well-being and adaptive functioning in older adults living alone (OALA).

**Methods:**

A randomized controlled trial was conducted with 22 participants (experimental *n* = 11; control *n* = 11). Primary outcomes were depressive symptoms, measured by the Geriatric Depression Scale-15 (GDS-15) and the Beck Depression Inventory-II (BDI-II), and adaptive behavior, measured by the Adaptive Behavior Assessment System-II (ABAS-II).

**Results:**

Compared with the control group, the experimental group demonstrated significant reductions in depressive symptoms (GDS-15 post-test mean difference = −9.45; BDI-II post-test mean difference = −15.82) and the General Adaptive Composite (GAC) of adaptive behavior significantly increased (post-test mean difference = 10.64). Significant improvements were observed in the Conceptual and Social domains, while no significant changes were found in the Practical domain. At the skill level, the experimental group demonstrated significant gains in Communication, Self-Direction, Social, and Community Use (*p* < 0.05), whereas the control group exhibited declines across multiple skills.

**Conclusion:**

Life Review Art Therapy effectively enhanced both emotional well-being and daily functioning in OALA, supporting its potential as a community-based, non-pharmacological intervention.

**Clinical trial registration:**

https://clinicaltrials.gov/study/NCT06763757, Unique Protocol ID: 202410001RINA.

## Introduction

Late-life depression and functional decline among older adults living alone (OALA) have emerged as critical global public health challenges, requiring the development of innovative and culturally sensitive interventions. Due to the compounded effects of aging, bereavement, and prolonged social isolation, OALA are particularly vulnerable to emotional distress, cognitive impairment, and social withdrawal (Hadida-Naus et al., 2023; McKay et al., 2021). Although global life expectancy continues to rise, mental health support for older populations remains inadequate. For instance, In Taiwan, the treatment rate for late-life depression is under 40% (Chang et al., 2022), with official data showing only 27% seeking care and 11% receiving effective treatment—well below rates in other developed countries (Ministry of Health and Welfare, 2022). This discrepancy underscores an urgent need for community-based, non-pharmacological interventions that simultaneously address psychological well-being and adaptive functioning.

*Life review therapy* (LRT), first conceptualized by Butler (1963), is grounded in reminiscence, a naturally occurring process in later life through which individuals recall both positive and negative memories to derive meaning (Westerhof and Slatman, 2019). Butler distinguished general reminiscence, defined as the recollection of personal memories, from life review, which entails a more evaluative examination of past events. LRT seeks to help older adults integrate life experiences, resolve conflicts, and achieve ego integrity. Meta-analyses have consistently demonstrated the effectiveness of LRT in alleviating depressive symptoms and enhancing life satisfaction (Lin et al., 2024; Pinquart, 2024). Beyond depression, studies have shown that LRT promotes psychological well-being, strengthens identity and social connectedness, and supports cognitive functioning (Yen and Lin, 2018). More recent evidence suggests that LRT may also help mitigate anxiety and trauma-related symptoms, underscoring its potential as a broader therapeutic approach (Lely and Kleber, 2022). However, questions remain regarding the cross-cultural applicability of LRT and its differential effects across diverse populations, which warrant further investigation (Choudhury et al., 2020; Hofer et al., 2017).

Despite these demonstrated benefits, the effectiveness and cultural adaptability of LRT remain subject to debate. Hofer et al. (2017) found that negative reminiscence was associated with impaired need satisfaction and increased depressive symptoms, highlighting cultural differences in how individuals process painful memories. Choudhury et al. (2020) similarly argued that LRT is not a one-size-fits-all intervention, as outcomes are shaped by cultural context and individual conditions such as social support and health status. In some cases, LRT may even evoke guilt, anxiety, or sadness when unresolved or traumatic memories are recalled (Keisari et al., 2023). Likewise, Cetinkol et al. (2020) demonstrated that lower acceptance of the past and higher death anxiety are significantly associated with geriatric depression. These findings suggest that cultural context plays a decisive role in shaping both the processes and outcomes of life review.

Taiwan provides a particularly compelling cultural context for understanding the complexities of life review. The island underwent Japanese colonial rule from 1895 to 1945, followed by nearly four decades of martial law under the Republic of China (1949–1987). This history fostered political fear, social repression, and a “culture of silence” that curtailed freedom of expression and suppressed emotional communication, leaving enduring psychological scars (National Human Rights Museum, 2021). These legacies, transmitted through cultural narratives and family storytelling, continue to shape attitudes toward memory, identity, and emotional expression (Huang, 2019). Moreover, many older Taiwanese adults carry childhood memories of war, poverty, resource scarcity, and enforced patriotism, which remain deeply embedded in their life stories (Chen et al., 2012).

As a result, Taiwan has developed into a multicultural society shaped by Chinese, Japanese, and Austronesian indigenous traditions. In later life, older adults often experience identity crises and a diminished sense of life meaning as they withdraw from primary social roles, particularly given their reliance on family and community resources (Kahana et al., 2014; World Health Organization, 2022, 2023a,b). This vulnerability heightens the risk of depression and social isolation. Against this backdrop, cultural sensitivity is crucial in the design and implementation of psychosocial interventions.

Beyond cultural and psychosocial factors, trauma and cognitive decline further complicate interventions for older adults living alone (OALA). According to the *Diagnostic and Statistical Manual of Mental Disorders, Fifth Edition* (DSM-5; American Psychiatric Association, 2013), trauma is defined as a stress response to overwhelming events, which can fragment autobiographical memory, weaken social connections, and intensify depressive symptoms (Rubin et al., 2008). This study is grounded in Trauma-Informed Art Therapy (TIAT), which emphasizes six principles including safety, trust, collaboration, empowerment, and cultural sensitivity (Substance Abuse and Mental Health Services Administration [SAMHSA], 2014). In art therapy, this approach prevents retraumatization and promotes meaning-making and regulation (Cloitre, 2020; Malchiodi, 2020).

Art therapy, through creative processes such as drawing and imagination, provides an alternative pathway for self-expression and healing when verbal communication is limited (Annous et al., 2022). Moreover, art therapy has been shown to effectively address complex emotional states such as guilt, sadness, and anxiety (Kaimal et al., 2019). Within a trauma-informed framework, it provides a safe medium to externalize suppressed emotions, reconstruct life narratives, and foster resilience (Malchiodi, 2020). Furthermore, art-making activities supported by photographs or symbolic objects can evoke meaningful memories, reinforce identity, and enhance social interaction (Matheson-Monnet, 2020). While not all older adults living alone meet clinical trauma criteria, many face cumulative adversities such as bereavement, poverty, or sociopolitical oppression (Huang, 2019). Thus, a trauma-informed perspective strengthens the rationale for integrating life review and art therapy while safeguarding cultural and psychological safety.

Importantly, converging reviews indicate that *executive function* (EF) is amenable to improvement through diverse interventions (Diamond and Ling, 2016). EF encompasses higher-order cognitive processes, including planning, initiation, execution, monitoring, and inhibition of behavior, playing a critical role in self-regulation and adaptive functioning in complex tasks (Menon and D’Esposito, 2022). EF is essential for maintaining autonomy in older adults but declines with age and is more severely impaired in late-life depression (Szymkowicz et al., 2023). Stress, grief, loneliness, and poor health further exacerbate this vulnerability (Diamond and Ling, 2016). Within this context, visual and expressive art therapy has demonstrated potential benefits for cognition, nonverbal emotional expression, and resilience, though the quality of evidence remains inconsistent and calls for more rigorous trials (Zhao and Rice, 2024). Collectively, these insights underscore the importance of developing integrative, non-pharmacological interventions that simultaneously support cognitive resilience, emotional expression, and trauma recovery in OALA.

Recent studies in East Asian contexts, including Taiwan, have demonstrated that life review can reduce depressive symptoms and improve life satisfaction, self-esteem, and social interactions, supporting its feasibility in these cultural settings (Tam et al., 2021). Evidence further suggests that cultural tailoring—by incorporating local language, values, and historical experiences—enhances both the acceptability and effectiveness of life review interventions (Diwan et al., 2023). In Taiwan, life review has been shown to improve life satisfaction (Lee et al., 2023) and group reminiscence can reduce depressive symptoms (Yao et al., 2020), while art therapy is increasingly applied in community and care settings (Jia and Tung, 2024; Yao, 2024). However, their integration has not yet been systematically examined, with existing reviews treating these approaches separately (McQuade and O’Sullivan, 2023; Pinquart, 2024).

In addition to evaluating efficacy, broader implementation frameworks such as RE-AIM and CFIR highlight the importance of adoption, sustainability, and contextual support (Racey et al., 2021; Safaeinili et al., 2019). Given that OALA face a “triple vulnerability” of aging, trauma, and isolation (Hadida-Naus et al., 2023), the present study implemented a culturally adapted life review art therapy (LRAT) program grounded in Taiwanese history and culture. Furthermore, to move beyond the predominant focus on psychological outcomes, we incorporated adaptive behavior measures (ABAS-II) to assess whether improvements in emotional well-being also translate into enhanced daily functioning. Together, these innovations informed the current trial, which aimed to evaluate both the psychological and functional effects of LRAT among older adults living alone in Taiwan.

This study aims to investigate the effects of a life review art therapy (LRAT) intervention on depressive symptoms and adaptive behaviors among OALA. The following hypotheses were tested: (1) Alleviate depressive symptoms; (2) Alleviate emotional distress in this population; (3) Improve adaptive function. By integrating reflective life review with creative art-based processes, this intervention seeks to empower older adults to reconstruct life narratives, enhance emotional regulation, and strengthen the executive and social skills necessary for independent living. Through this study, we aim to provide empirical evidence supporting LRAT as an effective, non-pharmacological approach that addresses the multidimensional needs of OALA.

## Materials and methods

### Study design

**Figure 1:**
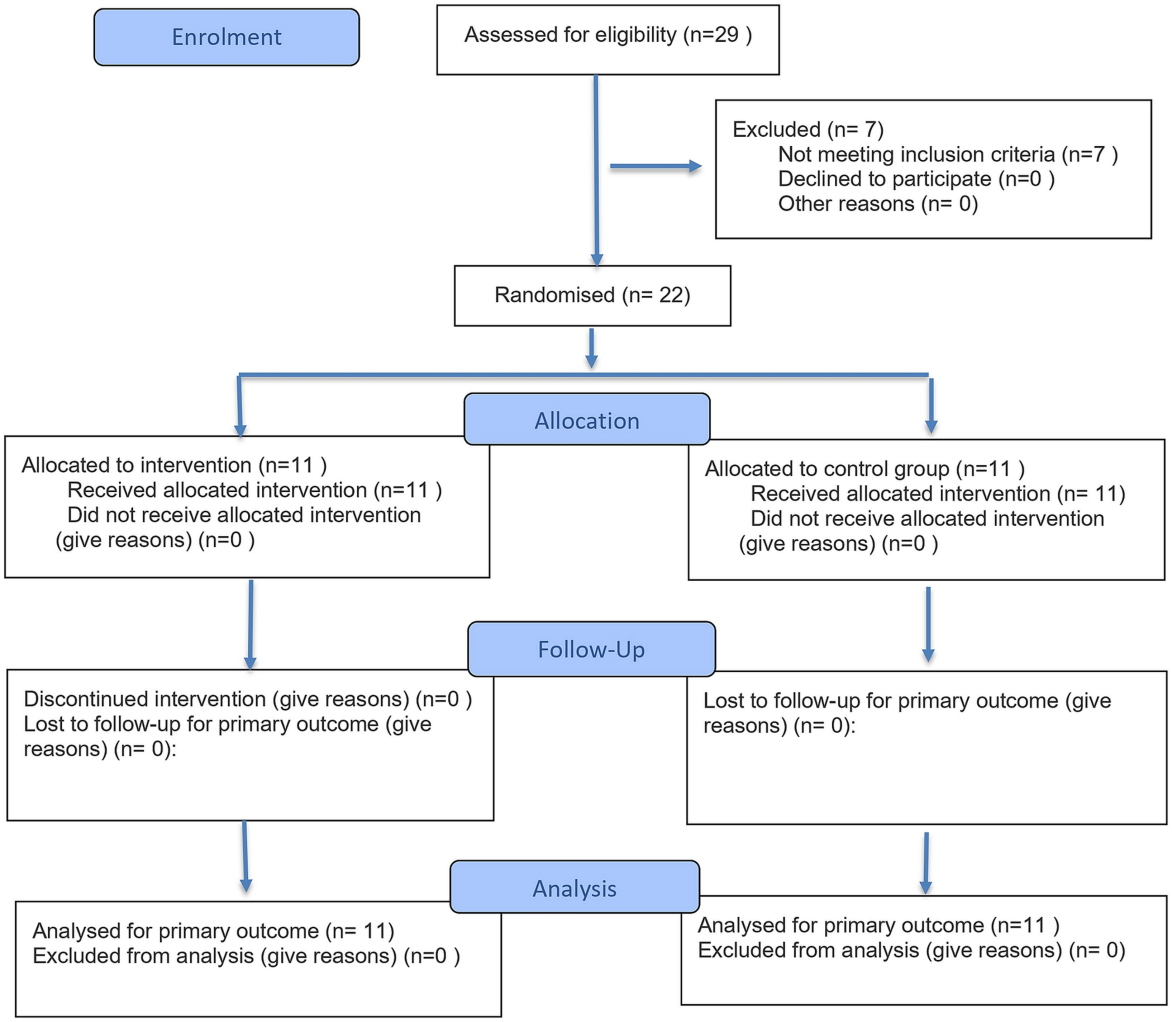
CONSORT 2025 flow diagram illustrating the recruitment and allocation process. This figure was created by the authors based on actual study data, detailing participant screening, random allocation, intervention completion, and final analysis to ensure transparency in reporting (Hopewell et al., 2025; Schulz et al., 2010).

A randomized controlled trial was conducted to evaluate the effects of the LRAT program on depression, adaptive behavior, and emotional distress among OALA over a 12-week period. The intervention was implemented between December 2024 and March 2025. The recruitment and allocation process is presented in Figure 1 (CONSORT flow diagram), which outlines participant screening, random allocation, intervention completion, and final analysis to ensure transparency in reporting (Hopewell et al., 2025; Schulz et al., 2010). In total, 22 participants were enrolled and randomly assigned to the intervention group (*n* = 11) or the control group (*n* = 11). All participants completed the study, with no dropout or loss to follow-up. This trial used a single-blind design (assessor-blinded); facilitators and participants could not be blinded (Boutron et al., 2008; Juul et al., 2021). The intervention was co-developed and delivered by licensed occupational therapists and trained research staff, while outcome assessments were administered by a separate team of blinded assessors to minimize measurement bias (Sterne et al., 2023; Schulz and Grimes, 2002).

### Eligibility criteria

Participants were eligible for inclusion if they met the following criteria: community-dwelling older adults aged ≥65 years who were able to communicate in Mandarin Chinese, Taiwanese, or English and provide written informed consent. In Taiwan, individuals aged ≥65 years are officially classified as older adults (National Development Council, 2024). To align with the study objective, participants were additionally required to be living alone, defined as residing in a single-person household without a spouse, partner, or other family members (Yeung, 2015), as this group is particularly vulnerable to social isolation and emotional distress (Tunstall, 1966/2024). Additional inclusion requirements included: no current use of psychotropic medications (e.g., for anxiety, depression, schizophrenia, or bipolar disorder); no prior participation in similar intervention studies; a Mini-Mental State Examination (MMSE) score of 24–30, indicating adequate verbal expression; no significant physical impairments that would hinder participation in drawing or other art-related activities; and no history of brain injury or psychiatric disorders. Consistent with a trauma-informed (rather than trauma-specific) approach, trauma exposure or diagnosis was not required for enrollment and was not used as a stratification criterion (Substance Abuse and Mental Health Services Administration [SAMHSA], 2014).

Participants were excluded if they had recently experienced major life events likely to destabilize emotional status (e.g., bereavement, severe illness, or financial hardship), exhibited strong resistance to art therapy, or were unable to complete the intervention sessions due to personal reasons. Further exclusion criteria included severe visual or hearing impairments that hindered effective participation, marked emotional dysregulation under stress (e.g., pronounced anxiety or emotional lability), or unwillingness to provide consent for audio recording during sessions. These exclusion criteria were intended to ensure participants’ adequate capacity and emotional stability for engagement, while minimizing potential confounds in evaluating intervention effects.

### Ethics approval and informed consent

The study protocol was approved by the NTUH-REC No.: 202410001RINA Institutional Review Board (IRB; approval number: NTUH-REC No.: 202410001RINA). All participants provided written informed consent prior to study enrollment, in accordance with ethical guidelines.

### Data collection procedure

Participants were recruited via posters, online ads, and flyers, and then randomized into experimental and control groups. Participants provided informed consent after receiving study information from occupational therapists and researchers at the Yunlin Branch of the National Taiwan University Hospital. Standardized outcome measures (ABAS-II, GDS-15, BDI-II) were administered by blinded research assistants; occupational therapists conducted baseline clinical screening (e.g., MMSE) and safety monitoring and did not administer outcome assessments. Interventions and assessments were conducted at a community center in Hsinchu City. Researchers, supervised by occupational therapists, managed the interventions, data collection, and analysis (Argyropoulos et al., 2015).

### Randomization

Participants were recruited from community centers in the East District of Hsinchu City and randomly assigned to the experimental (*n* = 11) or control (*n* = 11) group using a random number generator in Excel 2019 (Microsoft, Redmond, WA, United States), stratified by age and gender. A secondary generator (Research Randomizer, Version 4.0) was used to produce a list of 11 unique numbers, and participants whose identification numbers matched were assigned to the experimental group. Allocation was conducted by an independent research assistant using sequentially numbered, opaque, sealed envelopes (SNOSE) to ensure concealment and disclosed to facilitators only after pretesting (Higgins et al., 2019; Schulz et al., 2010). This study used an assessor-blinded (single-blind) design. To prevent contamination, participants were instructed not to share intervention materials or assessments; pre- and post-tests were scheduled on different dates, times, and in separate classrooms, and outcome assessments consisted of self-report measures administered by trained assistants blinded to group allocation to minimize assessor-related bias (Schulz et al., 2010). Assessors and analysts used de-identified group codes (e.g., A/B) for data handling and analyses until completion of the primary analysis.

### Intervention group

Grounded in Life Review Therapy (LRT; Butler, 1963) and adapted to the sociocultural context of older adults living alone in Taiwan (Hofer et al., 2017), the Life Review Art Therapy (LRAT) curriculum incorporated reflection on turning points, family, and identity through multimodal art-making (e.g., timeline, memory mapping, drawing, mask-making, collage) (Shin et al., 2023). Consistent with a trauma-informed (rather than trauma-specific) approach, the intervention emphasized safety, choice, empowerment, and cultural sensitivity (Substance Abuse and Mental Health Services Administration [SAMHSA], 2014). LRAT was delivered in 12 weekly sessions (90 min each) at a community center in Hsinchu City. Each cohort comprised 4 participants and was co-facilitated by one licensed occupational therapist and one trained research facilitator. Delivery adhered to a manualized protocol with fidelity checklists and attendance logs.

The LRAT intervention, based on Erikson’s ego integrity theory, facilitated life experience reconstruction through art and reflection (Butler, 1963; Erikson and Erikson, 1998). This 12-week program aimed to build resilience, reduce depressive cognitions, and enhance EF by addressing grief, loss, and trauma. Art therapy incorporates directive, non-directive, and hybrid approaches to improve self-expression, relationships, memory, and agency (Herulf Scholander et al., 2024). EF improvement was emphasized due to its influence on language, emotion, thought, and memory (Manning and Steffens, 2018; Szymkowicz et al., 2023). Unlike physical exercise–based EF interventions (Blomstrand et al., 2023), art therapy stimulates cognitive processes, enhancing communication, emotion articulation, and self-awareness (Davis et al., 2019). LRAT utilized art to foster past acceptance, EF enhancement, and trauma integration. A consolidated summary of the 10 intervention sessions is presented in Table 1, which outlines weekly themes, introductory prompts, core activities, and therapeutic focus. Twelve representative artworks and participants’ quotations are included as Supplementary material (see: Life Review Art Therapy [LRAT] Intervention Sessions). Representative artworks from Sessions 2, 3–4, and 7 are shown in Supplementary Figures 2–4.

**Table 1:** Life review art therapy (LRAT) intervention curriculum.

Session	Theme	Prompt	Activities	Therapeutic focus
1	Life Timeline and Orientation	“Create a timeline of your life.”	Timeline drawing; Group sharing	Orientation (Butler, 1963)
2	Childhood and Family Memories	“Draw a picture that represents a childhood memory.”	Event mapping; Family drawings	Memory (Malchiodi, 2020)
3	Protective Strengths	“If you were an animal, which would you be?”	Animal self-drawings	Empowerment (Decker et al., 2018)
4	Relational Needs	“Which animal represents your relationships?”	Relational animal drawings	Relationships (Herulf Scholander et al., 2024)
5	Self-Reflection and Ocean Collage	“Select a seashell; what does it mean to you?”	Seashell collage; Ocean symbolism	Reflection (Pinquart, 2024)
6	Coping and Resilience	“Imagine your journey as a boat on the sea.”	Ocean meditation; Journey drawings	Resilience (Lely and Kleber, 2022)
7	Inner and Outer Self	“Create a mask of your inner and outer sides.”	Mask-making (inner vs. outer self)	Identity (Cloitre, 2020)
8	Loss and Farewell	“Write/draw a letter to a loved one.”	Perspective drawing; Letter writing	Grief (Keisari et al., 2023)
9	Joy and Positive Resources	“Draw or paint a joyful memory.”	Music meditation; Free drawing	Joy (Shin et al., 2023)
10	Processing Complex Loss	“Depict circumstances of a personal loss.”	Grief collage artworks	Integration (Nelson et al., 2022)
11	Autobiographical Reflection	“Create a timeline representing emotional changes.”	Autobiographical timelines	Well-Being (Lin et al., 2024)
12	Course Reflection	“What does a happy life mean to you now?”	Group reflection; Verbal sharing	Closure (Erikson and Erikson, 1998)

### Session details

**Figure 2:**
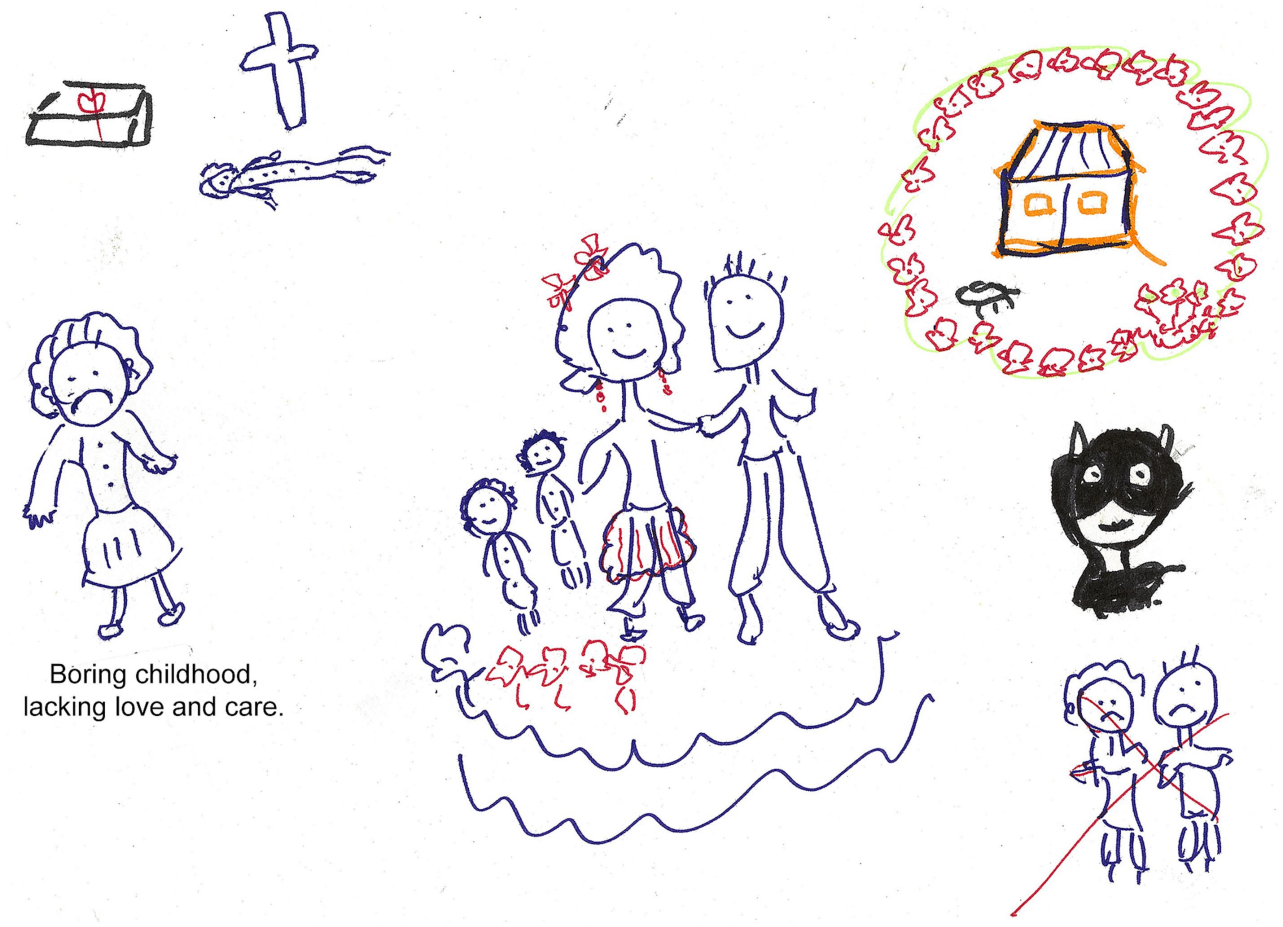
Life-review artwork depicting parental conflict. A participant portrayed conflicting feelings toward her parents—“a painful memory of actions that overshadowed my childhood”—illustrating the trauma-processing component of the intervention.

In Session 2, participants visually explored autobiographical memories by mapping salient life events and using artwork to externalize associated thoughts and emotions (see Figure 2).

**Figure 3:**
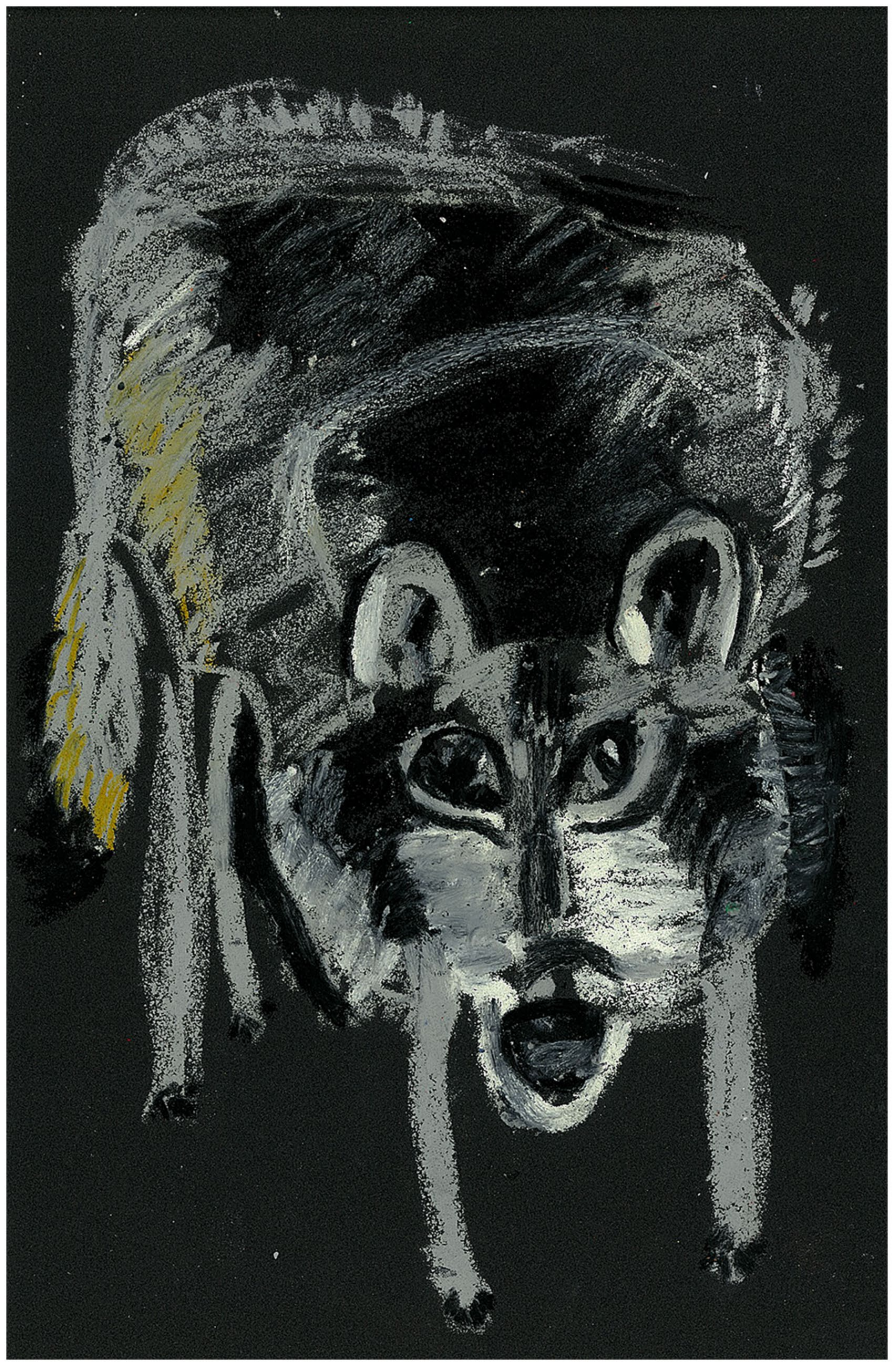
Animal symbolism—wolf as independence and self-protection. The participant noted, “Wolves represent independence, strength, and self-protection, reminding me to care for myself before helping others”.

Sessions 3–4: Participants created self-empowering images by drawing animals as extensions of themselves and external elements of their experiences. These exercises facilitated exploration of self-identity, protective strengths, and relational needs. A representative example is shown in Figure 3, illustrating how one participant identified with a wolf to symbolize independence and self-protection (see Supplementary Figure 1 for an additional example from Session 4).

**Figure 4:**
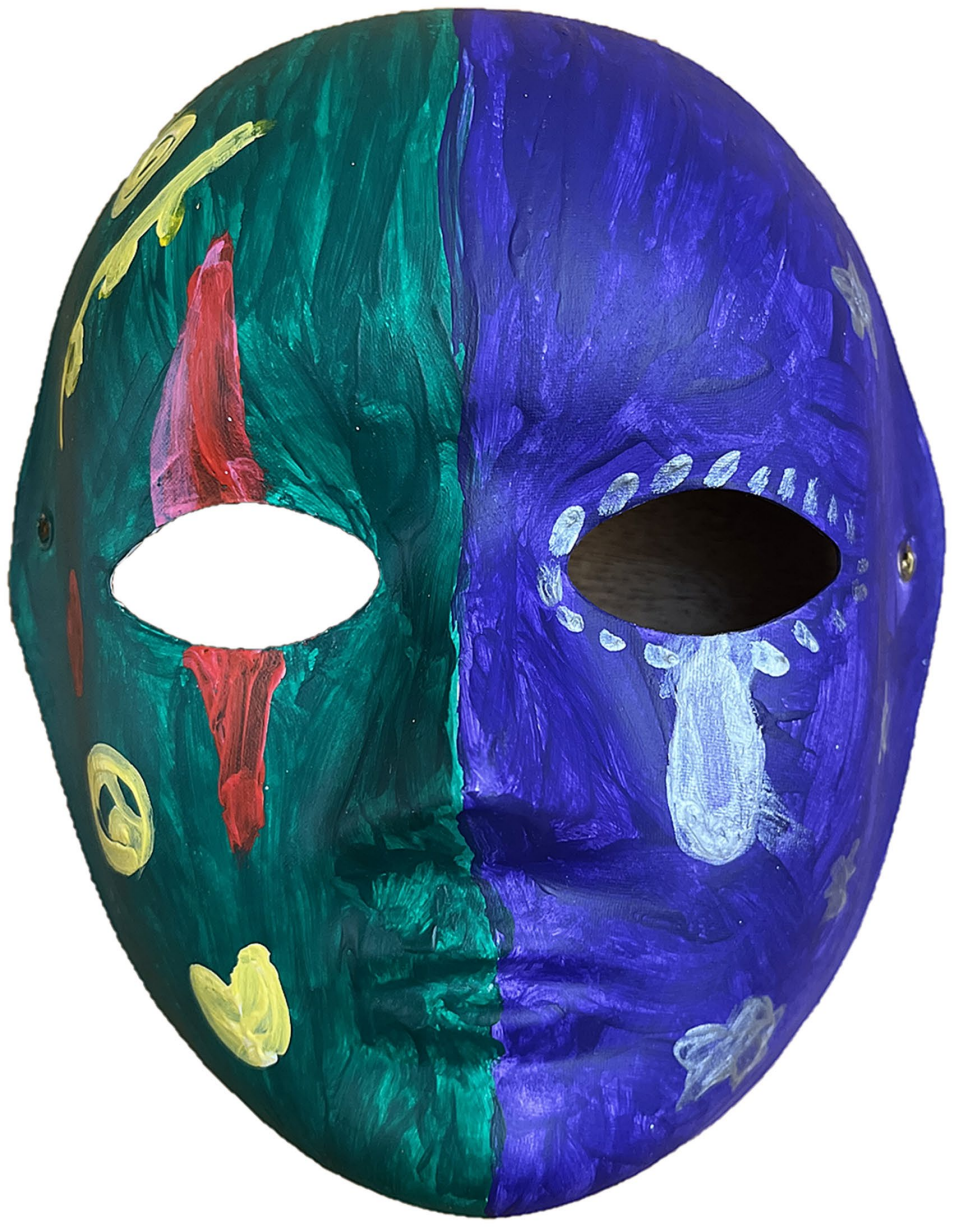
Mask-making to contrast inner and outer selves. One participant explained, “The purple reflects my inner pain and helplessness, in contrast with my outward desire for peace,” exemplifying trauma-informed identity integration.

In Session 7, mask-making was used to juxtapose inner experience with outward presentation, strengthening self-efficacy and coherence through trauma-informed reflection (see Figure 4).

### Control group

Although the control and experimental groups were administered during the same month and week, the control group did not receive LRAT. To minimize potential confounding variables, the control group continued to receive routine community services and maintain their existing social activities to enhance interpretability and external validity, consistent with the principles of pragmatic experimentation (Zwarenstein et al., 2008). This arrangement preserved psychosocial support for the control group while minimizing the risks of contamination and attrition due to unequal benefit, with delayed intervention offered when necessary to address ethical and retention considerations (Cunningham et al., 2013).

### Assessments and follow-up

Outcomes were assessed at weeks 1 and 12. In this study, no missing data were reported, and all participants completed both pretest and posttest assessments; thus, no data imputation procedures were necessary. This strengthens the validity of the study findings (Jakobsen et al., 2017).

### Outcome measures

The study instruments were categorized as follows:

1. Chinese version of the Adaptive Behavior Assessment System – Second Edition (ABAS-II). The ABAS-II (Harrison and Oakland, 2003), adapted for use with older adults, with specific forms designed for individuals aged 65–74 and 75–84 years. This instrument evaluates adaptive behavior through the General Adaptive Composite (GAC), three adaptive domains (Conceptual, Social, and Practical), and nine adaptive skill areas. The Conceptual domain includes Communication, Functional Academics, and Self-Direction; the Social domain includes Leisure and Social; and the Practical domain includes Community Use, Home Living, Health and Safety, and Self-Care. The GAC and adaptive domains are reported as standard scores (*M* = 100, SD = 15), whereas the adaptive skill areas are reported as scaled scores (*M* = 10, SD = 3). Data were collected through participant self-report, which captures subjective perceptions of functional status and complements the limitations of proxy ratings and objective tests. Self-report tools provide a practical and cost-effective means of assessing multidimensional daily functioning, particularly suitable for community-based research (Harrison and Oakland, 2015; Sandercock et al., 2020).
2. Chinese version of the Beck Depression Inventory – Second Edition (BDI-II). The Beck Depression Inventory-II (BDI-II) is a widely used self-report tool for depressive symptoms. Research has confirmed that the BDI-II possesses a two-factor structure and exhibits high applicability, reliability, and validity in community-dwelling older adults (Steer et al., 2000). This evidence supports the present study’s use of its affective and somatic subscales to distinguish between dimensions of depression. Furthermore, the BDI-II total score is used to classify symptom severity (minimal to severe) and has been validated for assessing and monitoring depression in this population (Segal et al., 2008).
3. Chinese version of the 15-item Geriatric Depression Scale (GDS-15). The GDS-15 is a validated tool for detecting depressive symptoms in cognitively intact older adults (≥65 years) (Park and Kwak, 2020; Vinkers et al., 2004). The 15-item scale categories scores as: <5 (normal), 5–9 (mild depression), and >10 (moderate to severe depression). Responses to items 2, 3, 4, 6, 8, 9, 10, 12, 14, and 15 (positive), or items 1, 5, 7, 11, and 13 (negative) indicate depressive symptoms.

A dual-scale approach was adopted to assess depressive symptoms. The BDI-II was employed to evaluate symptom severity and sensitivity to change over 2 weeks (Beck et al., 1996), while the GDS-15 served as a geriatric-friendly screen focusing on the past week and minimizing somatic confounds (Sheikh and Yesavage, 1986). Their complementary properties in timeframe, response format, and design rationale enhance both precision and feasibility in older adult populations.

### Statistical analysis

The analysis comprised three sections: descriptive statistics for participant profiling, pre- and post-test comparisons including normality checks, and hypothesis testing. Although normality is typically assumed in small samples (*n* = 22), potential estimation bias warrants consideration. In this study, normality was examined using skewness and kurtosis statistics. Except for daily living skills, most variables approximately followed a normal distribution. For the Chinese version of the ABAS-II, skewness and kurtosis values fell within the acceptable range of ±2 for communication (skewness = −0.133, kurtosis = −0.982), community use (skewness = 0.340, kurtosis = −0.368), functional academics (skewness = 0.880, kurtosis = 0.767), and health and safety (skewness = 0.402, kurtosis = −0.193), supporting the normality assumption. However, daily living skills in the pretest showed higher negative skewness (skewness = −1.361) and positive kurtosis (kurtosis = 2.363). Overall, the distributions of the other variables supported the validity of subsequent statistical analyses.

In this study, a pre-post experimental design with a small sample size (*N* = 22), Bayesian paired-sample t-test offers several advantages over traditional frequentist testing (van de Schoot et al., 2015). While the frequentist approach provides a *p*-value indicating the probability of the data under the null hypothesis (H₀), it cannot quantify evidence for H₀ or directly state the probability of the hypotheses themselves (Dienes, 2011). The Bayesian method, in contrast, calculates a Bayes Factor (BF) that directly quantifies the strength of evidence for the alternative hypothesis (H₁) over H₀. For instance, a BF₁₀ of 10 means the data are 10 times more likely under H₁ than H₀, a more intuitive interpretation of evidence (Wagenmakers et al., 2010).

Critically, Bayesian testing can provide affirmative evidence for the null hypothesis (e.g., BF₁₀ < 1), which is impossible with a non-significant *p*-value (Rouder et al., 2009). This is particularly valuable in small-sample studies where a lack of significance may stem from low power. Furthermore, the Bayesian approach is more robust to optional stopping, allowing for more flexible data analysis plans without inflating Type I error rates (Wagenmakers et al., 2012). By providing a continuous measure of evidence and enabling direct probabilistic statements about hypotheses, the Bayesian t-test offers a more nuanced and informative analysis for research with limited sample sizes.

A Bayes Factor (BF₀₁) greater than 1 supports the null hypothesis (H₀), while a value less than 1 supports the alternative (H₁). Evidence strength is categorized as follows: 1–3 is anecdotal, 3–10 is moderate, 10–30 is strong, 30–100 is very strong, and >100 is extreme evidence for the respective hypothesis. Table 2 provides guidance on interpreting BF values based on (Lee and Wagenmakers, 2013).

**Table 2:** Bayes factor judgment criteria.

BF₀₁ value	Evidence for H₀/H₁
>100	Extreme evidence for H_0_
30–100	Very strong evidence for H_0_
10–30	Strong evidence for H_0_
3–10	Moderate evidence for H_0_
1–3	Anecdotal evidence for H_1_
1	No evidence
1/3–1	Anecdotal evidence for H_1_
1/3–1/10	Moderate evidence for H_1_
1/10–1/30	Strong evidence for H_1_
1/30–1/100	Very strong evidence for H_1_
<1/100	Extreme evidence for H_1_

Criteria adapted from Lee and Wagenmakers (2013).

### Participant characteristics

The sample (*N* = 22) was equally divided between the contrast and experimental groups (50% each). Education levels varied, with high school graduation and elementary school being the most common (27.3% each). The majority of participants were aged 64–74 years old (81.8%), indicating a predominantly young-old adult sample.

## Results

Table 3 presents the results of Bayesian independent-samples *t*-tests comparing the experimental and control groups across psychological and somatic subscale outcomes. Descriptive statistics (means and SDs), mean differences, standard errors, BF₀₁, *t* values, degrees of freedom (df), and 95% credible intervals are reported. Note. Variables with the suffix “_p” indicate post-test scores.

**Table 3:** Results of Bayesian independent samples *t*-tests comparing experimental and control groups (*N* = 22).

Group statistics		Descriptive statistic		Posterior distribution for independent sample mean						
				Bayes factor independent sample test					95% credible interval	
Variable	Group	Mean	SD	Mean difference	Pooled SE difference	BF_01_	*t*	df	Lower	Upper
Depression	Control	8.00	2.65	2.55	1.065	0.404	2.390	20	0.32	4.77
	Experimental	10.55	2.34							
Depression_p	Control	12.09	1.58	−9.45	0.584	0.000	−16.203	20	−10.67	−8.24
	Experimental	2.64	1.12							
Affective symptoms	Control	9.64	5.16	3.64	2.091	1.034	1.739	20	−1.00	8.27
	Experimental	13.27	4.63							
Affective symptoms_p	Control	18.18	6.40	−15.82	1.959	0.000	−8.074	20	−20.18	−11.45
	Experimental	2.36	1.12							
Somatic	Control	3.64	2.11	1.73	0.682	0.320	2.534	20	0.21	3.24
	Experimental	5.36	0.81							
Somatic_p	Control	7.55	2.91	−4.09	0.902	0.007	−4.536	20	−6.10	−2.08
	Experimental	3.45	0.69						−1.00	8.27

Values represent group means and standard deviations. The suffix “_p” indicates post-test scores. SE = pooled standard error of the mean difference. BF₀₁ = Bayes factor (null vs. alternative hypothesis). CI = 95% credible interval. t = t statistic. df = degrees of freedom. Experimental group *n* = 11; control group *n* = 11.

For Depression–Depression_p (GDS-15), the experimental group (*M* = 10.55, SD = 2.34) scored higher than the control group (*M* = 8.00, SD = 2.65), with a mean difference of 2.55, *t*(20) = 2.39, *p* = 0.028, 95% CI [0.32, 4.77], BF₀₁ = 0.404, suggesting moderate evidence for the alternative hypothesis. At Depression_p, the experimental group’s mean decreased markedly to 2.64 (SD = 1.12), while the control group’s mean increased to 12.09 (SD = 1.58). The mean difference was −9.45, *t*(20) = −16.20, *p* < 0.001, 95% CI [−10.67, −8.24], BF₀₁ = 0.000, providing decisive evidence for the alternative hypothesis (BF₀₁ < 0.33).

For Affective–Affective_p (BDI-II), the experimental group scored higher (*M* = 13.27, SD = 4.63) than the control group (*M* = 9.64, SD = 5.16), with a mean difference of 3.64, *t*(20) = 1.74, *p* = 0.097, 95% CI [−1.00, 8.27], BF₀₁ = 1.034, suggesting anecdotal evidence for the null hypothesis. At Affective_p, the experimental group’s mean decreased substantially to 2.36 (SD = 1.12), while the control group’s mean increased to 18.18 (SD = 6.40). The resulting mean difference was −15.82, *t*(20) = −8.07, *p* < 0.001, 95% CI [−20.18, −11.45], BF₀₁ = 0.000, providing decisive evidence for the alternative hypothesis.

For Somatic–Somatic_p (BDI-II), the experimental group (*M* = 5.36, SD = 0.81) scored higher than the control group (*M* = 3.64, SD = 2.11), with a mean difference of 1.73, *t*(20) = 2.53, *p* = 0.020, 95% CI [0.21, 3.24], BF₀₁ = 0.320, suggesting substantial evidence for the alternative hypothesis. At Somatic_p, the experimental group’s mean decreased to 3.45 (SD = 0.69), whereas the control group’s mean increased to 7.55 (SD = 2.91). The mean difference was −4.09, *t*(20) = −4.54, *p* < 0.001, 95% CI [−6.10, −2.08], BF₀₁ = 0.007, providing very strong evidence for the intervention’s efficacy.

Table 4 presents the results of Bayesian related-samples *t*-tests comparing pre–post differences in the experimental and control groups across adaptive behavior domains and skills. Descriptive statistics include mean differences, standard errors, *t* values, degrees of freedom, *p* values, 95% confidence intervals, and Bayes factors (BF₀₁). Note. Variables with the suffix “_p” indicate post-test scores. The full detailed table is provided in the Supplementary material (see Table 4 Bayesian related-sample *t*-test results).

**Table 4:** Pre–post differences in ABAS-II general adaptive composite across domains and skills by group.

Group	Pretest–post-test	Posterior distribution for related-sample mean difference							
		Mean difference	SE mean	Bayes factor	*t*	df	*p*	95% CI	
								Lower	Upper
Experimental	General adaptive Composite General adaptive Composite_p	10.64	1.57	0.002	6.782	10	0.000	6.59	14.68
	Conceptual domain Conceptual domain_p	4.27	0.63	0.002	6.742	10	0.000	2.64	5.91
	Social domain Social domain_p	3.09	0.59	0.010	5.197	10	0.000	1.56	4.62
	Practical domain Practical domain_p	1.50	0.50	0.755	3.000	1	0.205	0.51	3.30
	Communication Communication_p	2.27	0.47	0.016	4.847	10	0.001	1.06	3.48
	Community Use Community Use_p	1.73	0.49	0.098	3.540	10	0.005	0.47	2.99
	Functional Academics Functional Academics_p	0.91	0.55	1.406	1.662	10	0.127	−0.50	2.32
	Home Living Home Living_p	0.18	0.38	4.019	0.482	10	0.640	−0.79	1.15
	Health and Safety Health and Safety_p	1.36	0.74	1.125	1.838	10	0.096	−0.55	3.28
	Leisure Leisure_p	0.64	0.73	3.160	0.872	10	0.404	−1.24	2.52
	Self_care–Self_care_p	−0.18	0.50	4.214	−0.363	10	0.724	−1.47	1.11
	Self-Direction Self-Direction_p	1.45	0.47	0.197	3.068	10	0.012	0.23	2.68
	Social Social_p	2.27	0.76	0.225	2.975	10	0.014	0.30	4.24
Control	General Adaptive Composite	−13.18	3.14	0.039	−4.196	10	0.002	−21.28	−5.08
	General Adaptive Composite_p								
	Conceptual domain	−5.91	0.86	0.001	−6.890	10	0.000	−8.12	−3.70
	Conceptual domain_p								
	Social domain Social domain_p	−3.36	0.75	0.027	−4.461	10	0.001	−5.31	−1.42
	Practical domain Practical domain_p	−4.36	1.85	0.552	−2.359	10	0.040	−9.13	0.41
	Communication Communication_p	−2.00	0.54	0.077	−3.708	10	0.004	−3.39	−0.61
	Community Use Community Use_p	−1.27	0.59	0.730	−2.160	10	0.056	−2.79	0.25
	Functional Academics Functional Academics_p	−1.45	0.37	0.053	−3.975	10	0.003	−2.40	−0.51
	Home Living Home Living_p	−1.18	0.46	0.420	−2.550	10	0.029	−2.38	0.01
	Health and Safety Health and Safety_p	−0.73	0.66	2.604	−1.099	10	0.298	−2.43	0.98
	Leisure Leisure_p	−0.82	0.35	0.580	−2.324	10	0.042	−1.73	0.09
	Self_care Self_care_p	−0.91	0.39	0.584	−2.319	10	0.043	−1.92	0.10
	Self-Direction Self-Direction_p	−2.45	0.25	0.000	−9.925	10	0.000	−3.09	−1.82
	Social Social_p	−2.36	0.54	0.031	−4.345	10	0.001	−3.77	−0.96

Values represent mean differences and standard errors from related-samples t tests (df = 10). The suffix “_p” indicates post-test scores. Bayesian evidence is reported as BF₀₁. CI = 95% confidence interval. ABAS-II = Adaptive Behavior Assessment System–Second Edition. Two-tailed *p*-values are reported (e.g., *p* < 0.001).

For General Adaptive Composite–General Adaptive Composite_p, the experimental group showed a significant increase (*M* = 10.64, *SE* = 1.57), *t*(10) = 6.78, *p* < 0.001, 95% CI [6.59, 14.68], BF₀₁ = 0.002, providing strong evidence for the alternative hypothesis. The control group demonstrated a significant decline (*M* = −13.18, *SE* = 3.14), *t*(10) = −4.20, *p* = 0.002, 95% CI [−21.28, −5.08], BF₀₁ = 0.039, also supporting the alternative hypothesis.

Within the Conceptual domain–Conceptual domain_p, the experimental group improved significantly (*M* = 4.27, *SE* = 0.63), *t*(10) = 6.74, *p* < 0.001, 95% CI [2.64, 5.91], BF₀₁ = 0.002, with strong evidence for the alternative hypothesis. Conversely, the control group declined significantly (*M* = −5.91, *SE* = 0.86), *t*(10) = −6.89, *p* < 0.001, 95% CI [−8.12, −3.70], BF₀₁ = 0.001. For Social domain–Social domain_p, the experimental group showed a significant increase (*M* = 3.09, *SE* = 0.59), *t*(10) = 5.20, *p* < 0.001, 95% CI [1.56, 4.62], BF₀₁ = 0.010. The control group declined significantly (*M* = −3.36, *SE* = 0.75), *t*(10) = −4.46, *p* = 0.001, 95% CI [−5.31, −1.42], BF₀₁ = 0.027. For Practical domain–Practical domain_p, the experimental group showed no significant change (*M* = 1.50, *SE* = 0.50), *t*(10) = 3.00, *p* = 0.205, 95% CI [0.51, 3.30], BF₀₁ = 0.755, indicating anecdotal evidence for the null hypothesis. By contrast, the control group declined significantly (*M* = −4.36, *SE* = 1.85), *t*(10) = −2.36, *p* = 0.040, 95% CI [−9.13, −0.41], BF₀₁ = 0.552.

At the skill level, the experimental group showed significant improvements in Communication (*M* = 2.27, SE = 0.47, *t*(10) = 4.85, *p* = 0.001, 95% CI [1.06, 3.48], BF₀₁ = 0.016) and Self-Direction (*M* = 1.45, SE = 0.47, *t*(10) = 3.07, *p* = 0.012, 95% CI [0.23, 2.68], BF₀₁ = 0.197). Significant gains were also observed in Social skills (*M* = 2.27, SE = 0.76, *t*(10) = 2.98, *p* = 0.014, 95% CI [0.30, 4.24], BF₀₁ = 0.225) and Community Use (*M* = 1.73, SE = 0.49, *t*(10) = 3.54, *p* = 0.005, 95% CI [0.47, 2.99], BF₀₁ = 0.098). By contrast, the control group demonstrated significant declines in Home Living (*M* = −1.18, SE = 0.46, *t*(10) = −2.55, *p* = 0.029, 95% CI [−2.38, −0.01], BF₀₁ = 0.420) and Self-care (*M* = −0.91, SE = 0.39, *t*(10) = −2.32, *p* = 0.043, 95% CI [−1.92, −0.10], BF₀₁ = 0.584) (see Table 4).

## Discussion

This study examined the efficacy of a 12-week Life Review Art Therapy (LRAT) program on depressive symptoms, adaptive behavior, and affective symptoms in older adults living alone (OALA). The findings provide robust support for all three hypotheses. LRAT effectively alleviated depressive symptoms, enhanced adaptive behavior, and reduced emotional distress, with particularly pronounced improvements in communication, social interaction, and self-direction.

These differentiated outcomes highlight the multidimensional nature of depression (Beck et al., 1996). The reduction in somatic symptoms suggests that embodied art-making may help alleviate the physical manifestations of low mood (Kaimal et al., 2019; Malchiodi, 2020), while the decrease in affective depression reflects improved regulation of sadness and anhedonia. The improvement in emotional distress further underscores the broader role of Life Review Art Therapy (LRAT) in mitigating the psychosocial strain associated with isolation and adversity among older adults living alone (Hadida-Naus et al., 2023; Tam et al., 2021). Analysis using the Adaptive Behavior Assessment System–Second Edition (ABAS-II) revealed significant improvements across multiple domains in the experimental group, whereas the control group demonstrated declines. These findings contribute to a more comprehensive understanding of LRAT’s impact on daily functioning in this population (Harrison and Oakland, 2003). Compared with traditional significance testing, Bayesian analysis provided more nuanced evidence of these effects, which is particularly valuable in small-sample contexts (Lee and Wagenmakers, 2013). Taken together, these results underscore the theoretical rationale for integrating life review with art therapy to address both emotional and functional domains (Pinquart and Forstmeier, 2012; Malchiodi, 2020; Ferguson et al., 2021), supporting its feasibility and therapeutic efficacy as a culturally relevant psychosocial intervention for this vulnerable group.

These findings align with previous research demonstrating that life review helps integrate experiences and alleviates depressive symptoms in older adults (Wilfling et al., 2023). Prior studies have also suggested that life review enhances cognitive function (Ferguson et al., 2021; Lima, 2023). In parallel, art therapy has been shown to support executive functions (EFs) and emotional expression, making it particularly beneficial for individuals with difficulty articulating traumatic memories (Manning and Steffens, 2018; Ma, 2020; Szymkowicz et al., 2023). Beyond efficacy, the present results suggest that adoption, sustainability, and contextual support are critical considerations for future implementation, consistent with prior frameworks such as RE-AIM and CFIR (Racey et al., 2021; Safaeinili et al., 2019). For example, cultural factors in Taiwan, including strong family involvement and collective decision-making (Chen et al., 2025), likely facilitated engagement and acceptance, highlighting the need to tailor interventions to local values and care systems. The present study extends these findings by confirming the therapeutic efficacy of combining life review with art therapy in improving emotional well-being and adaptive functioning among community-dwelling older adults.

Our observed reductions in depression and improvements in social, communication, and self-directed abilities align with mechanisms of trauma-informed art practices: nonverbal creation can reduce the physiological and emotional load of accessing traumatic memories, enhance body awareness and self-regulation, and enable narrative reorganization at a “controllable distance.” Symbolic media—such as masks—are used within trauma-informed art therapy to support self-representation and the integration of internal and external experience (Malchiodi, 2020), which is consistent with the feedback we recorded during the mask-making activity in Session 7 and with prior work showing that symbolic objects can evoke meaningful memories and reinforce identity (Matheson-Monnet, 2020). These trauma-informed processes suggest that the observed improvements were not only outcomes of reminiscence but also the result of embodied and symbolic modes of expression that facilitated meaning-making, resilience, and identity reconstruction.

The randomized controlled trial design provides strong evidence for the effectiveness of the intervention and reduces the risk of confounding variables. The choice of a no-intervention control group in this study aligns with established recommendations for early-stage evaluation of novel psychosocial interventions (Mohr et al., 2009). In psychological and art therapy research, this design is particularly appropriate because blinding of facilitators and participants is often infeasible in psychosocial trials, and the primary objective is to assess preliminary efficacy rather than to compare with other active treatments. The use of a no-intervention control group is justified by considerations of feasibility, ethical concerns, and the need to evaluate the pure effects of life review art therapy (LRAT) without confounding from other activities (Mohr et al., 2009). By employing a no-intervention control, the design effectively isolates the specific therapeutic effects of LRAT, minimizing interference from nonspecific social interactions or placebo effects (Decker et al., 2018). Moreover, such a design provides a clear baseline for understanding the unique contribution of LRAT in reducing depressive symptoms and enhancing adaptive functioning among older adults living alone (Annous et al., 2022). These methodological considerations strengthen the internal validity and scientific rigor of our findings while supporting their translational potential for broader community-based applications.

This study employed multiple art therapy techniques, including timeline construction, memory mapping, drawing, mask-making, and collage, to facilitate visual exploration and emotional expression. By integrating trauma-informed principles with life review, the Life Review Art Therapy (LRAT) program provided a safe and culturally sensitive medium for older adults to externalize emotions, reconstruct life narratives, and foster resilience. Clinically, these findings demonstrate the therapeutic potential of LRAT for enhancing communication, self-direction, and social participation among older adults living alone.

At the policy level, delivering LRAT through Taiwan’s Long-Term Care 2.0 “ABC” community network may strengthen accessibility and continuity of care, aligning program logistics with existing home- and community-based services (HCBS) infrastructure (Hsu and Chen, 2019). Furthermore, mapping LRAT outcomes to domains prioritized by the World Health Organization (WHO) Age-Friendly Cities and Communities framework—such as social participation and community support—could facilitate cross-site adoption and ensure long-term sustainability (World Health Organization, 2023a,b).

Future research should expand sample size, incorporate long-term follow-up, and investigate potential moderators such as gender, trauma history, and cultural background. These methodological refinements will enhance generalizability and support evidence-based implementation. Collectively, LRAT emerges not only as an effective intervention but also as a scalable and culturally adaptable model for community-based elder care.

Nevertheless, this study has several limitations. First, the sample size was small (*N* = 22), recruitment was limited to a single community center, and no *a priori* power analysis or demographic stratification was conducted, reducing representativeness and generalizability. Second, standardized trauma measures were not employed, such as the Life Events Checklist for DSM-5 (LEC-5; Weathers et al., 2013a) or the PTSD Checklist for DSM-5 (PCL-5; Weathers et al., 2013b); future studies should incorporate these validated instruments to examine whether trauma history moderates intervention effects. In addition, potential moderators such as age, gender, or educational level were not analyzed. Third, therapist effects and self-reported assessments may have introduced bias related to provider variability, cognitive status, or social desirability (Day et al., 2024; Sandercock et al., 2020). Fourth, only short-term follow-up was conducted, limiting conclusions about the durability of intervention effects (Stathi et al., 2022). Although Bayesian methods were applied to partially address small-sample limitations, external validity remains constrained. Future studies should recruit larger and more diverse samples, integrate multi-informant and standardized assessments, and extend follow-up periods to strengthen validity, generalizability, and applicability.

## Conclusion

This study demonstrated that a 12-week Life Review Art Therapy (LRAT) program significantly reduced depressive symptoms, enhanced adaptive behavior, and alleviated emotional distress among older adults living alone. As a culturally sensitive and trauma-informed, community-based intervention, LRAT improved communication, self-direction, and social participation, providing strong evidence for its therapeutic value as a non-pharmacological approach to geriatric care. Beyond clinical outcomes, LRAT shows promise as a scalable model for community elder care. Its alignment with Taiwan’s Long-Term Care 2.0 “ABC” network and the World Health Organization’s Age-Friendly Cities and Communities framework underscores its policy relevance and translational potential. Future studies should recruit larger and more diverse samples, employ active control groups, and extend follow-up periods to evaluate the durability of effects and potential moderators such as trauma history, gender, and cultural background. Overall, LRAT emerges not only as an effective intervention but also as a culturally adaptable and community-feasible model for promoting healthy aging.

## Data Availability

The original contributions presented in the study are included in the article/Supplementary material, further inquiries can be directed to the corresponding author.

## References

[ref1] American Psychiatric Association. (2013). Diagnostic and statistical manual of mental disorders. 5th Edn. Washington, DC: American Psychiatric Publishing.

[ref2] Annous N. Al-Hroub A. El Zein F. (2022). A systematic review of empirical evidence on art therapy with traumatized refugee children and youth. Front. Psychol. 13:811515. doi: 10.3389/fpsyg.2022.811515, PMID: 35707659 PMC9189733

[ref3] Argyropoulos K. Bartsokas C. Argyropoulou A. Gourzis P. Jelastopulu E. (2015). Depressive symptoms in late life in urban and semi-urban areas of South-West Greece: an undetected disorder? Indian J. Psychiatry 57, 295–300. doi: 10.4103/0019-5545.166617, PMID: 26600585 PMC4623650

[ref4] Beck A. T. Steer R. A. Brown G. K. (1996). Manual for the Beck depression inventory-II. San Antonio, TX: Psychological Corporation.

[ref5] Blomstrand P. Tesan D. Nylander E. M. Ramstrand N. (2023). Mind body exercise improves cognitive function more than aerobic- and resistance exercise in healthy adults aged 55 years and older – an umbrella review. Eur. Rev. Aging Phys. Act. 20:15. doi: 10.1186/s11556-023-00325-4 37558977 PMC10413530

[ref6] Boutron I. Moher D. Altman D. G. Schulz K. F. Ravaud P. (2008). Extending the CONSORT statement to randomized trials of non-pharmacologic treatment: explanation and elaboration. Ann. Intern. Med. 148, 295–309. doi: 10.7326/0003-4819-148-4-200802190-00008, PMID: 18283207

[ref7] Butler R. N. (1963). The life review: an interpretation of reminiscence in the aged. Psychiatry 26, 65–76. doi: 10.1080/00332747.1963.11023339, PMID: 14017386

[ref8] Cetinkol G. Bastug G. Kizil E. T. O. (2020). Poor acceptance of the past is related to depressive symptoms in older adults. GeroPsych 33, 191–199. doi: 10.1024/1662-9647/a000227

[ref9] Chang T.-Y. Liao S.-C. Chang C.-M. Wu C.-S. Huang W.-L. Hwang J.-J. . (2022). Barriers to depression care among middle-aged and older adults in Taiwan’s universal healthcare system. Lancet Reg. Health. 26:100501. doi: 10.1016/j.lanwpc.2022.100501, PMID: 36213135 PMC9535419

[ref10] Chen T. J. Li H. J. Li J. (2012). The effects of reminiscence therapy on depressive symptoms of Chinese elderly: study protocol of a randomized controlled trial. BMC Psychiatry 12:189. doi: 10.1186/1471-244X-12-189, PMID: 23126676 PMC3507717

[ref11] Chen D. R. Young Y. Shayya A. Perre T. O’Grady T. (2025). Cultural interplay in end-of-life care decisions: Comparing advance directive beliefs and preferences among adults in the U.S. and Taiwan. BMC Palliative Care 24:104. doi: 10.1186/s12904-025-01736-z 40234926 PMC11998129

[ref12] Choudhury T. K. John K. C. Garrett R. K. Stagner B. H. (2020). Considering psychodynamic therapy for older adults. Psychodynamic Psychiatry 48, 152–162. doi: 10.1521/pdps.2020.48.2.152, PMID: 32628580

[ref13] Cloitre M. (2020). ICD-11 complex post-traumatic stress disorder: simplifying diagnosis in trauma populations. Br. J. Psychiatry 216, 129–131. doi: 10.1192/bjp.2020.43, PMID: 32345416

[ref14] Cunningham J. A. Kypri K. McCambridge J. (2013). Exploratory randomized controlled trial evaluating the impact of a waiting list control design. BMC Med. Res. Methodol. 13:150. doi: 10.1186/1471-2288-13-150, PMID: 24314204 PMC4029562

[ref15] Davis E. Smith-Adcock S. Towns L. (2019). Experiences of elementary school counselors and students in using reality art therapy to address chronic conditions. Prof. Sch. Couns. 22:2156759X19870792. doi: 10.1177/2156759X19870792

[ref16] Day M. A. Ehde D. M. Bindicsova I. Jensen M. P. (2024). Understanding the role of therapist quality in accounting for heterogeneity of patient outcomes in psychosocial chronic pain treatments. J. Pain 25, 843–856. doi: 10.1016/j.jpain.2023.10.007, PMID: 37832902

[ref17] Decker K. P. Deaver S. P. Abbey V. Campbell M. Turpin C. (2018). Quantitatively improved treatment outcomes for combat-associated PTSD with adjunctive art therapy: randomized controlled trial. Art Ther. 35, 184–194. doi: 10.1080/07421656.2018.1540822

[ref18] Diamond A. Ling D. S. (2016). Conclusions about interventions, programs, and approaches for improving executive functions that appear justified and those that, despite much hype, do not. Dev. Cogn. Neurosci. 18, 34–48. doi: 10.1016/j.dcn.2015.11.005, PMID: 26749076 PMC5108631

[ref19] Dienes Z. (2011). Bayesian versus orthodox statistics: which side are you on? Perspect. Psychol. Sci. 6, 274–290. doi: 10.1177/1745691611406920, PMID: 26168518

[ref20] Diwan S. Eliazar A. Pham D. Fuentes M. (2023). Evaluation of a culturally adapted reminiscence therapy intervention: improving mood, family and community connectedness in Spanish- and Vietnamese-speaking older adults. Transcult. Psychiatry 60, 973–984. doi: 10.1177/13634615231191996, PMID: 37615171

[ref21] Erikson E. H. Erikson J. M. (1998). The life cycle completed (extended version). New York: W. W. Norton & Company.

[ref22] Ferguson H. J. Brunsdon V. E. A. Bradford E. E. F. (2021). The developmental trajectories of executive function from adolescence to old age. Sci. Rep. 11:1382. doi: 10.1038/s41598-020-80866-1, PMID: 33446798 PMC7809200

[ref23] Hadida-Naus S. Spector-Mersel G. Shiovitz-Ezra S. (2023). Alone in the shadow of terror: strategies and internal resources of older adults living alone in a continuous traumatic situation. Am. J. Orthopsychiatry 93, 188–197. doi: 10.1037/ort0000663 37036668

[ref24] Harrison P. L. Oakland T. (2003). Adaptive behavior assessment system–second edition (ABAS-II) manual. San Antonio, Texas: Harcourt Assessment.

[ref25] Harrison P. L. Oakland T. (2015). Adaptive behavior assessment system. 3rd Edn. Torrance, California: Western Psychological Services (WPS).

[ref26] Herulf Scholander L. Vikström S. Boström A. M. Josephsson S. (2024). Inquiring into conditions for engaging in narrative relations on a geriatric ward: how interpretation matters in everyday practices. Int. J. Qual. Stud. Health Well-Being 19:2367851. doi: 10.1080/17482631.2024.2367851, PMID: 38870415 PMC11177706

[ref27] Higgins J. P. Savović J. Page M. J. Elbers R. G. Sterne J. A. (2019). Assessing risk of bias in a randomized trial. In J. P. T. Higgins, J. Thomas, J. Chandler, M. Cumpston, T. Li, M. J. Page, V. A. Welch (Eds.), Cochrane handbook for systematic reviews of interventions, (*2nd* edn.) John Wiley & Sons. 205–228.

[ref28] Hofer J. Busch H. Poláčková Šolcová I. Tavel P. Kartner J. (2017). When reminiscence is harmful: the relationship between self-negative reminiscence functions, need satisfaction, and depressive symptoms among elderly people from Cameroon, the Czech Republic, and Germany. J. Happiness Stud. 18, 389–407. doi: 10.1007/s10902-016-9731-3

[ref29] Hopewell S. Chan A. W. Collins G. S. Hróbjartsson A. Moher D. Schulz K. F. . (2025). CONSORT 2025 statement: updated guideline for reporting randomised trials. BMJ 389:e081123. doi: 10.1136/bmj-2024-081123, PMID: 40228833 PMC11995449

[ref30] Hsu H.-C. Chen C.-F. (2019). LTC 2.0: the 2017 reform of home- and community-based long-term care in Taiwan. Health Policy 123, 912–916. doi: 10.1016/j.healthpol.2019.08.004, PMID: 31455563

[ref31] Huang C. H. (2019). Trauma, silence, and intergenerational memory in Taiwan. Taipei: Linking Publishing.

[ref32] Jakobsen J. C. Gluud C. Wetterslev J. . (2017). When and how should multiple imputation be used for handling missing data in randomised clinical trials – a practical guide with flowcharts. BMC Med. Res. Methodol. 17:162. doi: 10.1186/s12874-017-0442-1, PMID: 29207961 PMC5717805

[ref33] Jia L.-M. Tung F.-W. (2024). Effects of thematic art programs on cognitive function and depressive symptoms in older adults. Educ. Gerontol. 51, 925–941. doi: 10.1080/03601277.2024.2422335

[ref34] Juul S. Gluud C. Simonsen S. Frandsen F. W. Kirsch I. Jakobsen J. C. (2021). Blinding in randomised clinical trials of psychological interventions: a retrospective study of published trial reports. BMJ Evid. Based Med. 26:109. doi: 10.1136/bmjebm-2020-111407, PMID: 32998993

[ref35] Kahana E. Kelley-Moore J. A. Kahana B. (2014). Proactive approaches to successful aging: one clear path through the Forest. Gerontology 60, 466–474. doi: 10.1159/000360222, PMID: 24924437 PMC5493204

[ref36] Kaimal G. Jones J. P. Dieterich-Hartwell R. Acharya B. Wang X. (2019). Evaluation of long- and short-term art therapy interventions in an integrative care setting for military service members with post-traumatic stress and traumatic brain injury. Arts Psychother. 62, 28–36. doi: 10.1016/j.aip.2018.10.003

[ref37] Keisari S. Yaniv D. Gesser-Edelsburg A. Palgi Y. Neimeyer R. A. (2023). Meaning reconstruction 70 years later: processing older adults’ unfinished business in a drama therapy group. Psychotherapy 60, 573–586. doi: 10.1037/pst0000497, PMID: 37668568

[ref38] Lee M. D. Wagenmakers E.-J. (2013). Bayesian cognitive modeling: a practical course. Cambridge, United Kingdom: Cambridge University Press.

[ref39] Lee B.-O. Yao C.-T. Ramoo V. (2023). An evaluation of improving psychosocial life satisfaction among older adults in Taiwan Day care centers using life review work. J. Appl. Gerontol. 42, 842–851. doi: 10.1177/07334648221141408, PMID: 36437798

[ref40] Lely J. C. Kleber R. J. (2022). From pathology to intervention and beyond: reviewing current evidence for treating trauma-related disorders in later life. Front. Psych. 13:814130. doi: 10.3389/fpsyt.2022.814130, PMID: 35299824 PMC8921254

[ref41] Lima H. (2023). *How art therapy and the expressive therapies continuum can be used to enhance executive functioning skills: a literature review* [master’s thesis, Lesley university]. Lesley University DigitalCommons. Available online at: https://digitalcommons.lesley.edu/expressive_theses/710 (Accessed August 15, 2024).

[ref42] Lin J. Zhao R. Li H. Lei Y. Cuijpers P. (2024). Looking back on life: an updated meta-analysis of the effect of life review therapy and reminiscence on late-life depression. J. Affect. Disord. 347, 163–174. doi: 10.1016/j.jad.2023.11.050, PMID: 37995927

[ref43] Ma L. (2020). Depression, anxiety, and apathy in mild cognitive impairment: current perspectives. Front. Aging Neurosci. 12:9. doi: 10.3389/fnagi.2020.00009, PMID: 32082139 PMC7002324

[ref44] Malchiodi C. A. (2020). Trauma and expressive arts therapy: Brain, body, and imagination in the healing process. New York, NY: Guilford Publications.

[ref45] Manning K. J. Steffens D. C. (2018). State of the science of neural systems in late-life depression: impact on clinical presentation and treatment outcome. J. Am. Geriatr. Soc. 66, S17–S23. doi: 10.1111/jgs.15353, PMID: 29659005 PMC5905432

[ref46] Matheson-Monnet C. (2020). “Reminiscence therapy and intergenerational interventions for enhancing self-identity and social inclusion of older people and people living with dementia (PLDs)” in Handbook on promoting social justice in education. ed. Papa R. (Cham, Switzerland: Springer International Publishing). 687–710.

[ref47] McKay M. T. Cannon M. Chambers D. Conroy R. M. Coughlan H. Dodd P. . (2021). Childhood trauma and adult mental disorder: a systematic review and meta-analysis of longitudinal cohort studies. Acta Psychiatr. Scand. 143, 189–205. doi: 10.1111/acps.13268 33315268

[ref48] McQuade L. O’Sullivan R. (2023). Examining arts and creativity in later life and its impact on older people’s health and wellbeing: a systematic review of the evidence. Perspect. Public Health 144, 344–353. doi: 10.1177/17579139231157533, PMID: 36905227

[ref49] Menon V. D’Esposito M. (2022). The role of PFC networks in cognitive control and executive function. Neuropsychopharmacology 47, 90–103. doi: 10.1038/s41386-021-01152-w, PMID: 34408276 PMC8616903

[ref50] Ministry of Health and Welfare. (2022). NIH survey: 13% prevalence of depressive symptoms among middle-aged and older adults, but only 27% seek treatment, indicating lower care rates than in high-income countries such as Europe, the U.S., and Japan. Available online at: https://www.mohw.gov.tw/cp-16-71727-1.html (Accessed August 16, 2024).

[ref51] Mohr D. C. Spring B. Freedland K. E. Beckner V. Arean P. Hollon S. D. . (2009). The selection and design of control conditions for randomized controlled trials of psychological interventions. Psychother. Psychosom. 78, 275–284. doi: 10.1159/000228248, PMID: 19602916

[ref52] National Development Council. (2024). Population projections for the R.O.C. (Taiwan): 2022–2070. Retrieved December 5, 2024. Available online at: https://pop-proj.ndc.gov.tw/main_en/ (Accessed August 30, 2024).

[ref53] National Human Rights Museum (2021). Human rights under martial law in Taiwan. Taipei: Author.

[ref54] Nelson K. Lukawiecki J. Waitschies K. Jackson E. Zivot C. (2022). Exploring the impacts of an art and narrative therapy program on participants’ grief and bereavement experiences. OMEGA 90, 726–745. doi: 10.1177/00302228221111726, PMID: 35768193 PMC11528868

[ref55] Park S. H. Kwak M. J. (2020). Performance of the geriatric depression scale-15 with older adults aged over 65 years: an updated review 2000–2019. Clin. Gerontol. 44, 83–96. doi: 10.1080/07317115.2020.1839992, PMID: 33164674

[ref56] Pinquart M. (2024). Effects of reminiscence interventions on depression and anxiety: a meta-analysis of randomized controlled trials. Aging Ment. Health 28, 717–724. doi: 10.1080/13607863.2024.2320133 (PubMed: 38407110), PMID: 38407110

[ref9001] Pinquart M. Forstmeier S. (2012). Effects of reminiscence interventions on psychosocial outcomes: A meta-analysis. Aging Ment. Health. 16, 541–558. doi: 10.1080/13607863.2011.651434, PMID: 22304736

[ref57] Racey M. Markle-Reid M. Fitzpatrick-Lewis D. Usman M. A. Gagne H. Hunter S. . (2021). Applying the RE-AIM implementation framework to evaluate fall prevention interventions in community-dwelling adults with cognitive impairment: a review. BMC Geriatr. 21:407. doi: 10.1186/s12877-021-02344-3 34311700 PMC8314446

[ref58] Rouder J. N. Speckman P. L. Sun D. Morey R. D. Iverson G. (2009). Bayesian t tests for accepting and rejecting the null hypothesis. Psychonomic Bulletin and Review 16, 225–237. doi: 10.3758/PBR.16.2.225 19293088

[ref59] Rubin D. C. Berntsen D. Bohni M. K. (2008). A memory-based model of posttraumatic stress disorder: evaluating basic assumptions underlying the PTSD diagnosis. Psychol. Rev. 115, 985–1011. doi: 10.1037/a0013397, PMID: 18954211 PMC2762652

[ref60] Safaeinili N. Brown-Johnson C. Shaw J. G. Mahoney M. Winget M. (2019). CFIR simplified: pragmatic application of and adaptations to the consolidated framework for implementation research (CFIR) for evaluation of a patient-centered care transformation within a learning health system. Learn Health Sys. 3:e10201. doi: 10.1002/lrh2.10201 PMC6971122 31989028

[ref61] Sandercock R. K. Lamarche E. M. Klinger M. R. Klinger L. G. (2020). Assessing the convergence of self-report and informant measures for adults with autism spectrum disorder. Autism 24, 2256–2268. doi: 10.1177/1362361320942981, PMID: 32744068 PMC7541713

[ref62] Schulz K. F. Altman D. G. Moher D. Consort Group (2010). CONSORT 2010 statement: updated guidelines for reporting parallel group randomised trials. J. Clin. Epidemiol. 63, 834–840. doi: 10.1016/j.jclinepi.2010.02.005, PMID: 20346629

[ref63] Schulz K. F. Grimes D. A. (2002). Blinding in randomised trials: hiding who got what. Lancet 359, 696–700. doi: 10.1016/S0140-6736(02)07816-9, PMID: 11879884

[ref64] Segal D. L. Coolidge F. L. Cahill B. S. O’Riley A. A. (2008). Psychometric properties of the Beck depression inventory–II (BDI-II) among community-dwelling older adults. Behav. Modif. 32, 3–20. doi: 10.1177/0145445507303833 18096969

[ref65] Sheikh J. I. Yesavage J. A. (1986). Geriatric depression scale (GDS): recent evidence and development of a shorter version. Clin. Gerontol. 5, 165–173. doi: 10.1300/J018v05n01_09

[ref66] Shin E. Kim M. Kim S. Kim H. (2023). Effects of reminiscence therapy on quality of life and life satisfaction of the elderly in the community: a systematic review. BMC Geriatr. 23:420. doi: 10.1186/s12877-023-04001-1 37430198 PMC10332080

[ref67] Stathi A. Greaves C. J. Thompson J. L. Withall J. Ladlow P. Taylor G. . (2022). Effect of a physical activity and behaviour maintenance programme on functional mobility decline in older adults: the REACT (retirement in action) randomised controlled trial. Lancet Public Health 7:e316–e326. doi: 10.1016/S2468-2667(22)00004-4, PMID: 35325627 PMC8967718

[ref68] Steer R. A. Rissmiller D. J. Beck A. T. (2000). Use of the Beck depression inventory-II with depressed geriatric inpatients. Behav. Res. Ther. 38, 311–318. doi: 10.1016/S0005-7967(99)00068-6, PMID: 10665163

[ref69] Sterne J. A. C. Savović J. Page M. J. Elbers R. G. Higgins J. P. T. (2023). Chapter 8: assessing risk of bias in a randomized trial in Cochrane handbook for systematic reviews of interventions (version 6.5). (Eds). Higgins J. P. T. Thomas J. Chandler J. Cumpston M. Li T. Page M. J. . (Cochrane). Available online at: https://www.training.cochrane.org/handbook

[ref70] Substance Abuse and Mental Health Services Administration. (2014). SAMHSA’S concept of trauma and guidance for a trauma-informed approach (HHS publication no. SMA 14–4884). Rockville, MD: Substance Abuse and Mental Health Services Administration.

[ref72] Szymkowicz S. M. Gerlach A. R. Homiack D. Sanchez L. A. Chavez B. D. Kumar A. . (2023). Biological factors influencing depression in later life: role of aging processes and treatment implications. Transl. Psychiatry 13:160. doi: 10.1038/s41398-023-02464-9 37160884 PMC10169845

[ref73] Tam W. W. S. Poon S. N. Mahendran R. Kua E. H. Wu X. V. (2021). The effectiveness of reminiscence-based intervention on improving psychological well-being in cognitively intact older adults: a systematic review and meta-analysis. Int. J. Nurs. Stud. 114:103847. doi: 10.1016/j.ijnurstu.2020.103847, PMID: 33352435

[ref75] Tunstall J. (1966/2024). Old and alone: A sociological study of old people. New York, NY; London, UK: Routledge.

[ref77] van de Schoot R. Broere J. J. Perryck K. H. Zondervan-Zwijnenburg M. van Loey N. E. (2015). Analyzing small data sets using Bayesian estimation: the case of posttraumatic stress symptoms following mechanical ventilation in burn survivors. Eur. J. Psychotraumatol. 6:5216. doi: 10.3402/ejpt.v6.25216, PMID: 25765534 PMC4357639

[ref78] Vinkers D. J. Gussekloo J. Stek M. L. Westendorp R. G. van der Mast R. C. (2004). The 15-item geriatric depression scale (GDS-15) detects changes in depressive symptoms after a major negative life event: the Leiden 85-plus study. Int. J. Geriatr. Psychiatry 19, 80–84. doi: 10.1002/gps.1043, PMID: 14716703

[ref79] Wagenmakers E.-J. Lodewyckx T. Kuriyal H. Grasman R. (2010). Bayesian hypothesis testing for psychologists: a tutorial on the savage–dickey method. Cogn. Psychol. 60, 158–189. doi: 10.1016/j.cogpsych.2009.12.001 20064637

[ref80] Wagenmakers E.-J. Wetzels R. Borsboom D. van der Maas H. L. J. (2012). An agenda for purely confirmatory research. Perspect. Psychol. Sci. 7, 632–638. doi: 10.1177/1745691612463078 26168122

[ref81] Weathers F. W. Blake D. D. Schnurr P. P. Kaloupek D. G. Marx B. P. Keane T. M. (2013a). The life events checklist for DSM-5 (LEC-5) – Standard [measurement instrument]: National Center for PTSD, U.S. Department of Veterans Affairs. Available online at: https://www.ptsd.va.gov/ (Accessed August 16, 2024).

[ref82] Weathers F. W. Litz B. T. Keane T. M. Palmieri P. A. Marx B. P. Schnurr P. P. (2013b). The PTSD checklist for DSM-5 (PCL-5) – Standard [measurement instrument]: National Center for PTSD, U.S. Department of Veterans Affairs. Available online at: https://www.ptsd.va.gov/ (Accessed August 16, 2024).

[ref83] Westerhof G. J. Slatman S. (2019). In search of the best evidence for life review therapy to reduce depressive symptoms in older adults: a meta-analysis of randomized controlled trials. Clin. Psychol. Sci. Pract. 26:11. doi: 10.1111/cpsp.12301, PMID: 41069135

[ref84] Wilfling D. Calo S. Dichter M. N. Meyer G. Möhler R. Köpke S. (2023). Non-pharmacological interventions for sleep disturbances in people with dementia. Cochrane Database Syst. Rev. 1:CD011881. doi: 10.1002/14651858.CD011881.pub2, PMID: 36594432 PMC9808594

[ref85] World Health Organization (2022). National programmes for age-friendly cities and communities: A guide to development and implementation. Geneva, Switzerland: World Health Organization.

[ref86] World Health Organization (2023a). National programmes for age-friendly cities and communities: A guide and living toolkit: World Health Organization.

[ref87] World Health Organization (2023b). Progress report on the United Nations decade of healthy ageing, 2021–2023. Geneva: WHO.

[ref88] Yao C.-T. (2024). Effects of expressive arts therapy on cognitive function and depression among older adults with MCI in Taiwan. Educ. Gerontol. 50, 762–773. doi: 10.1080/03601277.2024.2336398

[ref89] Yao C.-T. Yang Y.-P. Lin C.-J. Liu H.-Y. (2020). Effect of group reminiscence therapy on depression and perceived meaning of life of veterans diagnosed with dementia at veteran homes. Soc. Work Health Care 59, 75–90. doi: 10.1080/00981389.2019.1710320, PMID: 31944912

[ref90] Yen H.-Y. Lin L.-J. (2018). A systematic review of reminiscence therapy for older adults in Taiwan. J. Nurs. Res. 26, 138–150. doi: 10.1097/jnr.0000000000000233, PMID: 29016468

[ref91] Yeung W.-J. J. (2015). Living alone: one-person households in Asia. Demogr. Res. 32, 1099–1112. doi: 10.4054/DemRes.2015.32.40

[ref92] Zhao R. Rice K. (2024). Exploring uses of visual arts-based interventions for mental health of marginalized populations: a scoping review. Arts & Health. 16, 1–19. doi: 10.1080/17533015.2024.2355134 38755973

[ref93] Zwarenstein M. Treweek S. Gagnier J. J. Altman D. G. Tunis S. Haynes B. . (2008). Improving the reporting of pragmatic trials: an extension of the CONSORT statement. BMJ 337:a2390. doi: 10.1136/bmj.a2390, PMID: 19001484 PMC3266844

