# Supplementary material for: A randomized controlled trial study of a life review art intervention for older adults living alone
Source: Front Psychol. 2025 Nov 19;16:1669119. doi: 10.3389/fpsyg.2025.1669119 (PMC12673666; doi:10.3389/fpsyg.2025.1669119)
Supplement: Supplementary file 1 [file Supplementary_file_1.pdf]

Life Review Art Therapy (LRAT) Intervention Sessions

Session	details
1	Participant created a timeline that illustrated emotional changes throughout their lives.
2	Participants visually explored memories by mapping significant events and using artwork to express emotions, thoughts, and past experiences. One participant depicted a painful memory of her parents: "Portrait of my parents, with deep emotional conflict due to past actions that ruined my childhood."
Figure 2	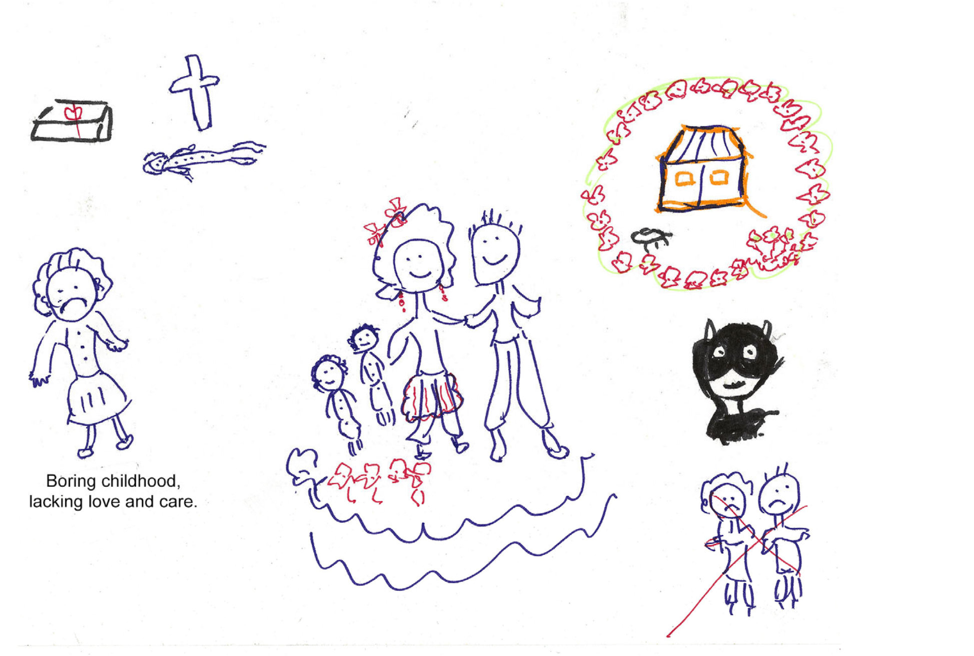

3	Participants created self-empowering images by drawing animals as extensions of themselves and external elements of their experiences. These exercises helped them explore strategies for finding comfort within themselves. In Figure 3, one participant admired wolves, stating: “Wolves represent independence, strength, and self-protection, teaching me to care for myself before helping others.” Another participant
Figure 3	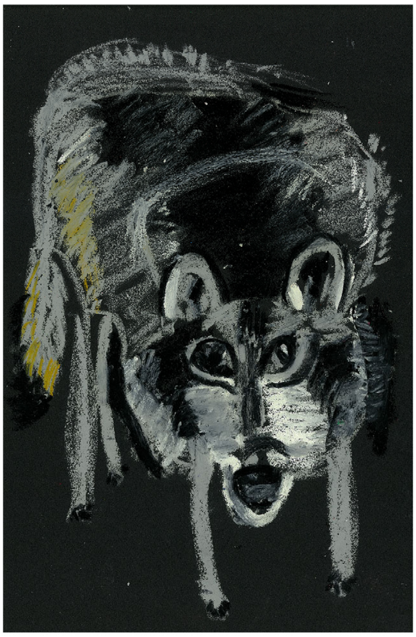
4	Participants explored self-identity through animal drawings, expressing emotional connections to their chosen animals. In Figure 4, identified with a crab, symbolizing his need for strong personal connections and a desire to build meaningful relationships.
Figure 4	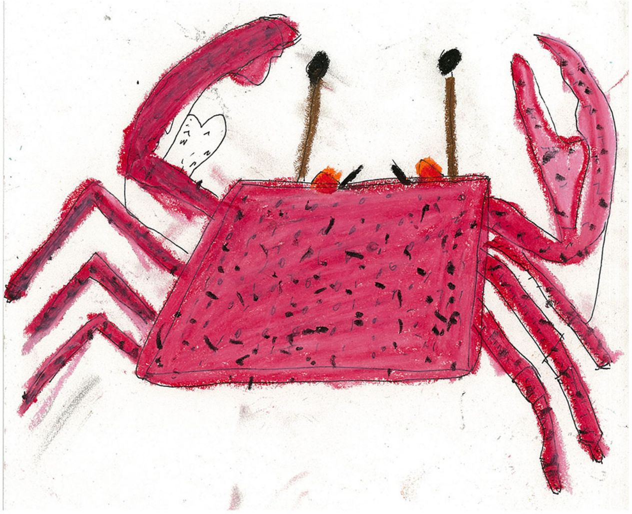

5	Participants engaged in a self-reflection journey by selecting a seashell and creating a collage, using their kinesthetic, perceptual, and cognitive senses to explore personal connections to the ocean. This process helped them identify and express emotions, leading to deeper insights into the symbolic significance of these ocean-related experiences. (Figure 5). Participants' collages from Session 5 represented ocean-related experiences and associated emotions. One participant described a joyful collage containing images symbolizing collective happiness.
Figure 5.	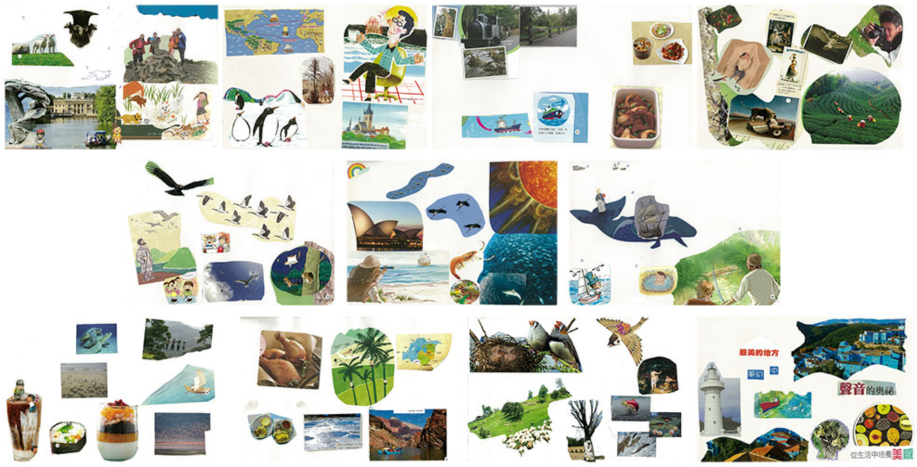

6	Participants meditated on ocean imagery, listened to ocean sounds, and visualized personal ocean memories. They freely expressed their emotions and shared their reflections with the group (Figure 6). Artwork from Session 6 offered poignant insights into emotional experiences. One participant depicted a small boat adrift at sea, symbolizing loneliness and vulnerability, yet conveying hope and resilience, and expressing gratitude for the spiritual guidance felt throughout the journey.
Figure 6	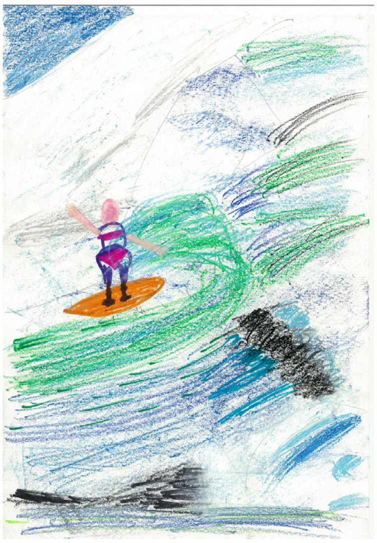
7	Participants created masks to visually represent the effects of trauma, including stress and invisible wounds. This artistic process promoted self-efficacy and a coherent sense of self (Figure 7). The mask served as a representation of inner and outer selves. One participant shared: “The color purple symbolizes my inner pain and helplessness, contrasting with my outward desire for peace.”
Figure 7	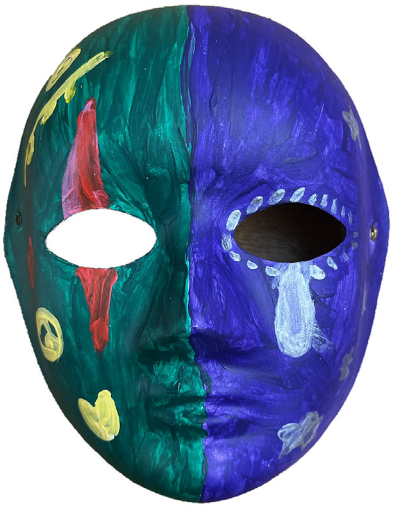

8	Participants adopted an observer’s perspective to describe events, fostering emotional distance and a sense of safety. They explored different forms of love, shared experiences, and reflected on positive memories. To process personal loss, they created drawings and wrote letters to loved ones, conveying heartfelt messages (Figure 8). Participants recorded traumatic experiences from an observer’s perspective and shared them with the group. One participant reflected on the sudden loss of a loved one in a car accident, contemplating grief and healing.
Figure 8	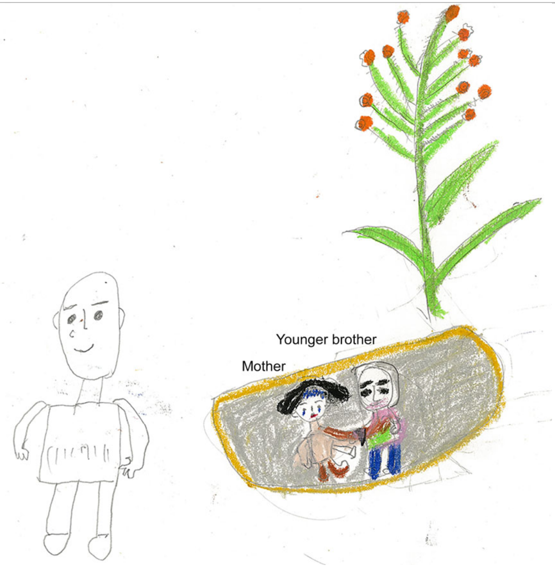
9	Participants listened to music and meditated on memory, using tactile media (e.g., crayons and paints) for free association and fluid conceptual shifts. These ideas were then integrated into cohesive stories (Figure 9). Artwork from Session 9 captured participants’ memories. One participant shared: “Cycling around the lake brings me joy and relaxation, regardless of my physical condition.”
Figure 9.	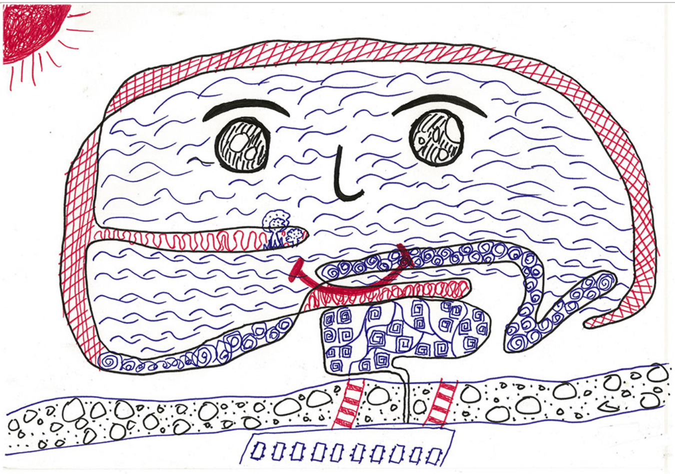

<table>
<tr><td>10</td><td>Participants created collages to process complex emotions associated with loss. They depicted their experiences, including the timing and circumstances of the loss, and shared their creations with the group (Figure 10). One participant reflected: “Remembering a loved one who was passionate about history, travel, and cultures, and cherishing their memory.”</td></tr>
<tr><td>Figure 10.</td><td>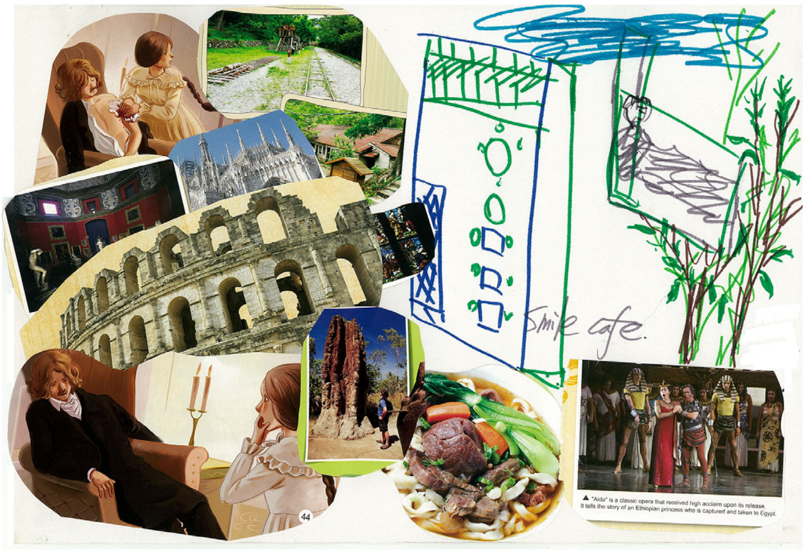
</td></tr>
<tr><td>11</td><td>Participants created autobiographical timelines using lines, images, and expressions to represent emotional changes. Sharing their life stories fostered self-awareness and positively impacted their mental well-being (Figure 11). A participant reflected: “Through life’s challenges, I’ve learned to cherish what I have, embracing its ups and downs while creating a peaceful home for my family.”</td></tr>
<tr><td>Figure 11.</td><td>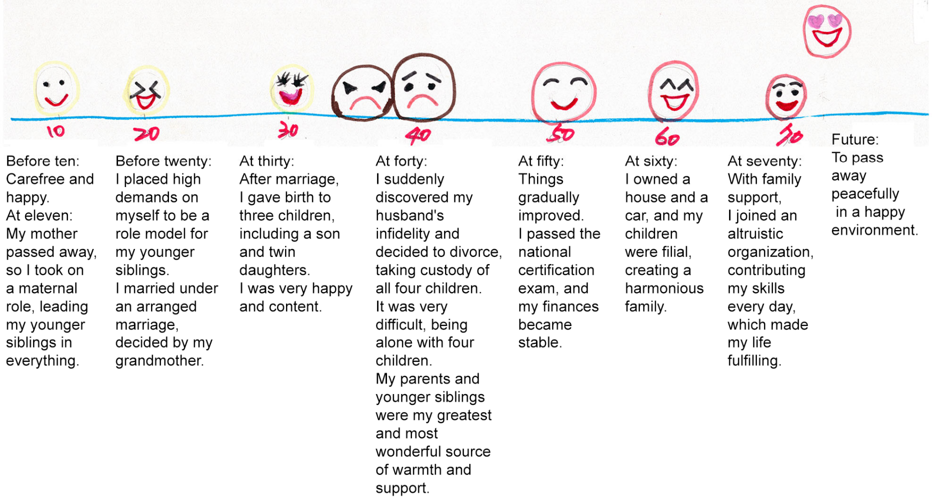
</td></tr>
</table>

12	Finally, participants shared their reflections on completing the 11-week course. One participant expressed: “I carry wounds from the past, but they have become blessings, bringing me joy.” They also found fulfillment in the painting process: “I feel like I’ve learned to take care of myself in my later years. That’s amazing! Keep going! Continue learning as long as I live!” When defining a happy life, another participant shared: “I know that’s me, exuding a faint fragrance, growing lush branches and leaves under the sunlight, a perfect embodiment of my inner and outer harmony.”
