# Supplementary material for: A randomized controlled trial study of a life review art intervention for older adults living alone
Source: Front Psychol. 2025 Nov 19;16:1669119. doi: 10.3389/fpsyg.2025.1669119 (PMC12673666; doi:10.3389/fpsyg.2025.1669119)
Supplement: Supplementary file 2 [file Supplementary_file_2.pdf]

The Supplementary Materials (ABAS-II GAC)

At the skill level, the experimental group improved significantly in **Communication–Communication_p** (*M* = 2.27, *SE* = 0.47), *t*(10) = 4.85, *p* = .001, 95% CI [1.06, 3.48], BF_{01} = 0.016, and **Self_Direction–Self_Direction_p** (*M* = 1.45, *SE* = 0.47), *t*(10) = 3.07, *p* = .012, 95% CI [0.23, 2.68], BF_{01} = 0.197, with moderate evidence for the alternative hypothesis. **Functional Academics–Functional Academics_p** did not change significantly (*M* = 0.91, *SE* = 0.55), *t*(10) = 1.66, *p* = .127, 95% CI [−0.50, 2.32], BF_{01} = 1.406, providing anecdotal evidence for the null hypothesis. For **Social–Social_p**, the experimental group improved significantly (*M* = 2.27, *SE* = 0.76), *t*(10) = 2.98, *p* = .014, 95% CI [0.30, 4.24], BF_{01} = 0.225. In contrast, **Leisure–Leisure_p** did not change significantly (*M* = 0.64, *SE* = 0.73), *t*(10) = 0.87, *p* = .404, 95% CI [−1.24, 2.52], BF_{01} = 3.160, supporting the null hypothesis. For **Community_Use–Community_Use_p**, the experimental group showed a significant increase (*M* = 1.73, *SE* = 0.49), *t*(10) = 3.54, *p* = .005, 95% CI [0.47, 2.99], BF_{01} = 0.098. The control group showed a non-significant decline (*M* = −1.27, *SE* = 0.59), *t*(10) = −2.16, *p* = .056, 95% CI [−2.79, 0.25], BF_{01} = 0.730. For **Home Living–Home Living_p**, the experimental group did not change significantly (*M* = 0.18, *SE* = 0.38), *t*(10) = 0.48, *p* = .640, 95% CI [−0.79, 1.15], BF_{01} = 4.019, supporting the null hypothesis. By contrast, the control group declined significantly (*M* = −1.18, *SE* = 0.46), *t*(10) = −2.55, *p* = .029, 95% CI [−2.38, −0.01], BF_{01} = 0.420. For **Health and Safety–Health and Safety_p**, the experimental group showed a non-significant increase (*M* = 1.36, *SE* = 0.74), *t*(10) = 1.84, *p* = .096, 95% CI [−0.55, 3.28], BF_{01} = 1.125. The control group also did not change significantly (*M* = −0.73, *SE* = 0.66), *t*(10) = −1.10, *p* = .298, 95% CI [−2.43, 0.98], BF_{01} = 2.604. For **Self_care–Self_care_p,** the experimental group did not change significantly (*M* = −0.18, *SE* = 0.50), *t*(10) = −0.36, *p* = .724, 95% CI [−1.47, 1.11], BF_{01} = 4.214. In contrast, the control group declined significantly (*M* = −0.91, *SE* = 0.39), *t*(10) = −2.32, *p* = .043, 95% CI [−1.92, −0.10], BF_{01} = 0.584.

Table 4.

Pre–Post Differences in ABAS-II General Adaptive Composite Across Domains and Skills by Group

Group	Pretest–post-test	Posterior distribution for related-sample mean difference							
		Mean Difference	SE Mean	Bayes Factor	t	df	p	95% CI	
								Lower	Upper
Experimental	General Adaptive Composite<br>General Adaptive Composite_p	10.64	1.57	0.002	6.782	10	0.000	6.59	14.68
	Conceptual domain<br>Conceptual domain_p	4.27	0.63	0.002	6.742	10	0.000	2.64	5.91

	Social domain	3.09	0.59	0.010	5.197	10	0.000	1.56	4.62
	Social domain_p								
	Practical domain	1.50	0.50	0.755	3.000	1	0.205	0.51	3.30
	Practical domain _p								
	Communication	2.27	0.47	0.016	4.847	10	0.001	1.06	3.48
	Communication_p								
	Community Use	1.73	0.49	0.098	3.540	10	0.005	0.47	2.99
	Community Use_p								
	Functional Academics	0.91	0.55	1.406	1.662	10	0.127	−0.50	2.32
	Functional Academics _p								
	Home Living	0.18	0.38	4.019	0.482	10	0.640	−0.79	1.15
	Home Living_p								
	Health and Safety	1.36	0.74	1.125	1.838	10	0.096	−0.55	3.28
	Health and Safety _p								
	Leisure	0.64	0.73	3.160	0.872	10	0.404	−1.24	2.52
	L~~L~~eisure_p								
	Self_c~~C~~are– Self_c~~C~~are_p	−0.18	0.50	4.214	−0.363	10	0.724	−1.47	1.11
	Self-Direction	1.45	0.47	0.197	3.068	10	0.012	0.23	2.68
	Self-Direction_p								
	Social	2.27	0.76	0.225	2.975	10	0.014	0.30	4.24
	Social_p								
Control	General Adaptive Composite	−13.18	3.14	0.039	−4.196	10	0.002	−21.28	−5.08
	General Adaptive Composite_p								
	Conceptual domain	−5.91	0.86	0.001	−6.890	10	0.000	−8.12	−3.70
	Conceptual								

domain_p								
Social domain Social domain_p	−3.36	0.75	0.027	−4.461	10	0.001	−5.31	−1.42
Practical domain Practical domain _p	−4.36	1.85	0.552	−2.359	10	0.040	−9.13	0.41
Communication	−2.00	0.54	0.077	−3.708	10	0.004	−3.39	−0.61
Communication_p								
Community Use	−1.27	0.59	0.730	−2.160	10	0.056	−2.79	0.25
Community Use_p								
Functional Academics	−1.45	0.37	0.053	−3.975	10	0.003	−2.40	−0.51
Functional Academics _p								
Home Living	−1.18	0.46	0.420	−2.550	10	0.029	−2.38	0.01
Home Living_p								
Health and Safety	−0.73	0.66	2.604	−1.099	10	0.298	−2.43	0.98
Health and Safety _p								
Leisure	−0.82	0.35	0.580	−2.324	10	0.042	−1.73	0.09
Leisure_p								
Self_care	−0.91	0.39	0.584	−2.319	10	0.043	−1.92	0.10
Self_care_p								
Self-Direction	−2.45	0.25	0.000	−9.925	10	0.000	−3.09	−1.82
Self-Direction_p								
Social	−2.36	0.54	0.031	−4.345	10	0.001	−3.77	−0.96
Social_p								

Note. Values represent mean differences and standard errors from related-samples t tests (df = 10). The suffix "_p" indicates post-test scores. Bayesian evidence is reported as BF_{01}. CI = 95% confidence interval. ABAS-II = Adaptive Behavior Assessment System–Second Edition. Two-tailed p values are reported (e.g., $p < .001$).
